# Metabolic Profiling-Based Evaluation of the Fermentative Behavior of *Aspergillus oryzae* and *Bacillus subtilis* for Soybean Residues Treated at Different Temperatures

**DOI:** 10.3390/foods9020117

**Published:** 2020-01-22

**Authors:** Hyejin Hyeon, Cheol Woo Min, Keumok Moon, Jaeho Cha, Ravi Gupta, Sang Un Park, Sun Tae Kim, Jae Kwang Kim

**Affiliations:** 1 Division of Life Sciences and Bio-Resource and Environmental Center, College of Life Sciences and Bioengineering, Incheon National University, Incheon 22012, Korea; 2 Department of Plant Bioscience, Life and industry Convergence Research Institute, Pusan National University, Miryang 50463, Korea; min0685@naver.com; 3 Department of Microbiology, College of Natural Sciences, Pusan National University, Busan 46241, Korea; moonko81@nate.com (K.M.); jhcha@pusan.ac.kr (J.C.); 4 Microbiological Resource Research Institute, Pusan National University, Busan 46241, Korea; 5 Department of Botany, School of Chemical and Life Science, Jamia Hamdard, New Delhi 110062, India; dr.ravigupta@jamiahamdard.ac.in; 6 Department of Crop Science, Chungnam National University, 99 Daehak-ro, Yuseong-gu, Daejeon 34134, Korea; supark@cnu.ac.kr

**Keywords:** soybean substrate, fermentation, *Aspergillus oryzae*, *Bacillus subtilis*, extraction temperature, metabolic profiling, multivariate analysis

## Abstract

Soybean processing, e.g., by soaking, heating, and fermentation, typically results in diverse metabolic changes. Herein, multivariate analysis-based metabolic profiling was employed to investigate the effects of fermentation by *Aspergillus oryzae* or *Bacillus subtilis* on soybean substrates extracted at 4, 25, or 55 °C. As metabolic changes for both *A. oryzae* and *B. subtilis* were most pronounced for substrates extracted at 55 °C, this temperature was selected to compare the two microbial fermentation strategies, which were shown to be markedly different. Specifically, fermentation by *A. oryzae* increased the levels of most organic acids, γ-aminobutyric acid, and glutamine, which were ascribed to carbohydrate metabolism and conversion of glutamic acid into GABA and glutamine. In contrast, fermentation by *B. subtilis* increased the levels of most amino acids and isoflavones, which indicated the high activity of proteases and β-glucosidase. Overall, the obtained results were concluded to be useful for the optimization of processing steps in terms of nutritional preferences.

## 1. Introduction

Although soybeans (*Glycine max* L. Merill) contain numerous functional components such as proteins, isoflavones, fatty acids, vitamins, and essential amino acids, their direct use is hindered by indigestion caused by the presence of anti-nutrients such as oligosaccharides and trypsin inhibitors [1]. Consequently, soybeans are usually subjected to processing (by heating, soaking, or fermentation), which results in the release of numerous metabolites.

Soaking in water, generally used as the initial step of soybean curd, soy sauce, and soy milk production, increases the levels of amino acids and vitamins [2] and releases functional proteins and isoflavones into soybean exudates, particularly when high water temperatures are employed [3]. A previous study reported that incubating in warm water (50 °C) resulted in a significant release of bioactive proteins (Kunitz trypsin inhibitor and Bowman-Birk protease inhibitor), which have inhibitory effects on various forms of cancer [4]. Furthermore, soaking at 60 °C elevated the extraction of isoflavones (genistein and daidzein), known as phytoestrogens, from soybeans [3]. However, the soybean residues remaining after extraction still contained some valuable nutrients. For instance, soaking at 60 °C increased the total polyphenol content (which has antioxidant potency) in soybeans [5], and extraction at 50 °C significantly improved amino acid composition [6]. However, the contained nutrients can be negatively affected by the use of excessively high temperatures and long extraction times, e.g., boiling reduces the levels of some vitamins and essential amino acids [7]. Therefore, an appropriate extraction temperature is important for efficiently using soybean residues as functional resources as well as extracting exudates from soybeans.

Following soaking and heating, fermentation by microbial enzymes such as amylase, glucosidase, and lipase is a promising method of soybean substrate hydrolysis, resulting in the destruction of plant cell walls and the liberation of various antioxidants to increase the levels of amino acids, tocopherols, and isoflavones [8]. Thus, the fermented soybeans are commonly consumed as functional foods especially in Asia. The fermented soybean products can be classified into several types according to fermenter species [9]. *Aspergillus oryzae*, a filamentous fungus, is widely exploited for the production of Meju, Duenjang, and soy sauce because of its low cost, high productivity, and safety [10,11], while *Bacillus subtilis* is widely used to produce Natto, Douchi, and Thua-nao because of its low pathogenicity and fast growth in inexpensive substrates [12]. For the above reasons, *A. oryzae* and *B. subtilis* are commonly selected as fermenters to produce various soybean products.

Metabolic profiling of fermented products can be used to monitor metabolic changes and evaluate product nutritional value and functional characteristics [13]. Although changes in fermented soybean metabolites are largely induced by different activities of fermenters (*A. oryzae* and *B. subtilis*), most prior investigations probed the individual effects of inoculated microbes on fermented soybeans, with only few studies comparing the effects of soybean fermentation by *A. oryzae* and *B. subtilis* on the corresponding metabolic profiles [12,14,15]. In addition, although microbial fermentations usually interact with substrate composition, no studies have dealt with the optimization of substrate extraction temperature (which influences metabolite contents) to maximize the levels of functional metabolites and antioxidants [16,17]. Thus, investigations comparing fermenter performances at different substrate extraction temperatures are urgently required. The present comparison study will be useful for identifying the differential nutritional quality of variously treated samples.

Herein, we perform a comprehensive metabolic comparison of soybeans fermented by *A. oryzae* and *B. subtilis* after water extraction at 4, 25, or 55 °C, using gas chromatography-time-of-flight mass spectrometry (GC-TOF-MS), gas chromatography-flame ionization detection (GC-FID), gas chromatography–mass spectrometry (GC–MS), and ultra-performance liquid chromatography-quadrupole time-of-flight mass spectrometry (UPLC-QTOF-MS) to identify primary and secondary metabolites. Metabolic differences are characterized by multivariate analysis, and correlation analysis is employed to explain sample variations and relationships between metabolites. The acquired data are matched with the metabolic pathways to investigate relative compositional fluctuations in fermented soybeans.

## 2. Materials and Methods 

### 2.1. Sample Preparation

The *A. oryzae* strain KCTC6983 was grown on yeast/peptone/dextrose agar at 25 °C for seven days, and the mycelium was collected into phosphate-buffered saline containing 0.01% Tween 80 and adjusted to an optical density of unity at 620 nm (OD620). The *B. subtilis* strain 168 was pre-cultured in tryptic soy broth at 37 °C overnight, inoculated into the same to a concentration of 1 vol%, and grown at 37 °C for 3 h. Subsequently, the culture was adjusted to an OD600 of unity.

Soybean (*Glycine max* L. Merill; 100 g) seeds were soaked in sterile water (300 mL) and hydrolyzed at 70 rpm and 4, 25, or 55 °C for 24 h using a modification of a previously reported method [4]. The hydrolyzed soybeans were harvested, lyophilized, and ground using a food mixer. The soybean medium was prepared by addition of the obtained soybean powder (10 g) to water (100 mL) and 15-min autoclaving at 121 °C. The spore suspension (1 × 10^7^ spores/mL) of *A. oryzae* (AO) or the cultured cells (2 × 10^8^ /mL) of *B. subtilis* (BS) was then inoculated into the soybean medium to a final concentration of 1 vol%. *A. oryzae* and *B. subtilis* were cultured at 30 °C for seven days or at 37 °C for three days in a shaking incubator, respectively. After cultivation, the medium was lyophilized. Overall, six types of fermented soybeans were obtained.

### 2.2. Extraction of Hydrophilic Compounds and Their Analysis by GC-TOF-MS

Hydrophilic compounds, including organic acids, amino acids, sugars, sugar alcohols, and amines, were extracted as described elsewhere [18]. Aliquots (10 mg) of lyophilized samples were extracted with methanol:water:chloroform (l mL; 2.5:1:1, *v/v/v*), and ribitol (60 μL, 0.2 mg/mL in methanol) was added as an internal standard (IS). After 30-min incubation at 37 °C and 1200 rpm, the samples were centrifuged at 16,000 × *g* and 4 °C for 3 min. The supernatants (800 μL) were transferred into new tubes, diluted with water (400 μL), and centrifuged under identical conditions. The resulting supernatants (900 μL) were transferred to new tubes and concentrated on a rotary evaporator (CC-105, TOMY, Tokyo, Japan) over 3 h. The residues were lyophilized for at least 16 h at −80 °C (MCFD8512 freeze-drier, IlShinBioBase, Dongducheon, Republic of Korea), and the dried samples were methoximated with 80 μL of methoxyamine hydrochloride in pyridine (2%, *w/v*) at 30 °C and 1200 rpm for 90 min. For silylation, the samples were incubated with 80 μL of *N*-methyl-*N*-(trimethylsilyl)trifluoroacetamide (MSTFA) at 37 °C and 1200 rpm for 30 min. Hydrophilic compounds were analyzed on an Agilent 6890N GC (Agilent, Atlanta, GA, USA) instrument equipped with a Pegasus TOF-MS (LECO, St Joseph, MI, USA) spectrometer. The extracted samples (1 μL) were injected in split mode (1:25 ratio) at an inlet temperature of 230 °C. Helium was used as a carrier gas at a flow rate of 1.00 mL/min. Metabolite separations were conducted for 28 min using a CP-Sil 8CB low-bleed/MS fused-silica capillary column (30 m × 0.25 mm, 0.25 μm; Agilent, Palo Alto, CA, USA). The initial oven temperature of 80 °C was held for 2 min, increased by 15 °C/min to 320 °C, and held for 10 min. Ion source and transfer line temperatures were set to 230 and 250 °C, respectively. *m/z* values of 85–600 were scanned, and the detector voltage was set to 1700 V. The acquired mass spectral data were compared with in-house, NIST, and Wiley9 libraries using ChromaTOF software (version 4.5, LECO, St Joseph, MI, USA). For quantification, the analyte/IS peak area ratio was estimated for selected ions (Table S1).

### 2.3. Extraction of Fatty Acids and Their Analysis by GC-FID 

Fatty acids were extracted using a modification of a previously reported method [19]. Each lyophilized sample (10 mg) was dissolved in 2.5 mL of chloroform:methanol (2:1, *v/v*), and pentadecanoic acid in chloroform (1 mg/mL, 0.1 mL) was introduced as an IS. After mixing, samples were extracted by 20-min ultrasonication, treated with aqueous NaCl (2.5 mL; 0.58%, *w/v*), and centrifuged at 4 °C and 13,000× *g* for 5 min. The bottom layers were transferred into new tubes and concentrated, and the residues were treated with methanol (0.18 mL), toluene (0.1 mL), and 5 M aqueous NaOH (0.02 mL). The resulting mixtures were incubated in a thermomixer (model 5355, Eppendorf AG, Hamburg, Germany) at 85 °C and 300 rpm for 5 min, treated with methanolic BF_3_ (14 vol%, 0.3 mL), processed as described above, and treated with water (0.4 mL) and pentane (0.8 mL). The obtained solutions were centrifuged at 4 °C and 750× *g* for 15 min, and supernatants were collected into new 2-mL tubes before concentration. Finally, solutions of the thus prepared fatty acid methyl esters (FAMEs) in hexane (0.1 mL) were passed through 0.5-µm syringe filters and subjected to GC-FID analysis (Agilent 7890B, Agilent, Atlanta, GA, USA). For each run, 1-µL samples were injected by an Agilent G4513A autosampler using a split ratio of 10:1. Nitrogen gas was flowed into a DB-Wax (30 m × 0.25 mm, 0.25 μm; Agilent) column at a rate of 1.00 mL/min. Both front inlet and detector temperatures were set to 250 °C. The initial GC temperature of 130 °C was held for 3 min, increased to 230 °C at 20 °C/min, further increased to 250 °C at 3 °C/min, and held for 5 min. Data were acquired using ChemStation software (Agilent), and qualification and quantification were conducted by comparison with FAME mixture standards (Table S2).

### 2.4. Extraction of Secondary Lipophilic Compounds and Their Analysis by GC–MS

Secondary lipophilic compounds such as policosanols, tocopherols, and sterols were extracted as described elsewhere [20]. Lyophilized samples (10 mg) were transferred to 15-mL tubes and extracted with ethanolic ascorbic acid (0.1%, *w/v*; 3 mL). The extracts were treated with 5α-cholestane (10 μg/mL in hexane, 50 μL) as an IS, vortexed, and placed in a water bath held at 85 °C for 5 min. Saponification with aqueous KOH (120 μL, 80% *w/v*) was conducted in a water bath held at 85 °C for 10 min and was followed by immediate cooling in ice for 5 min. The mixtures were treated with hexane and water (1.5 mL each), agitated, and centrifuged at 4 °C and 1200× *g* for 5 min. The supernatants were transferred into new tubes, and extraction was repeated with hexane (1.5 mL). The supernatants (3 mL) were blown dry with N_2_ gas and further concentrated on a rotary evaporator (TOMY). The residues were derivatizated with MSTFA (30 μL) and pyridine (30 μL) at 60 °C and 1200 rpm for 30 min, and the thus obtained lipophilic compounds were analyzed on a GC–MS QP2010 Ultra system equipped with an AOC-20i autosampler (GC–MS, Shimadzu, Kyoto, Japan). For separation, 1-μL sample aliquots were injected into an Rtx-5MS column (30 m × 0.25 mm, 0.25 μm; Restek, Bellefonte, PA, USA) at a split ratio of 1:10. Helium was flowed at a constant rate of 1.00 mL/min, and the inlet temperature equaled 290 °C. The initial oven temperature of 150 °C was maintained for 2 min, ramped to 320 °C at 15 °C/min, and held for 10 min. The ion source and interface temperatures equaled 230 and 250 °C, respectively. Spectra were acquired for *m/z* 85–600, and ions were detected in selected ion monitoring (SIM) mode for peak analysis. For absolute quantification, accurate calibration curves were determined for each lipophilic standard at loadings of 0.025–5.00 μg and fixed to an IS weight of 0.50 μg (Table S3). Chromatographic data were processed using Labsolutions GCMSsolution software (version 4.11, Shimadzu).

### 2.5. Extraction of Raffinose and Its Analysis by GC–MS

Raffinose was extracted using the protocol employed for hydrophilic compounds, and the extracts were injected into the GC–MS instrument (Shimadzu). The operating conditions were as follows: carrier gas = helium (1.00 mL/min); injection volume = 1 μL; split mode ratio = 25:1; injection temperature = 290 °C; column = Rtx-5MS (30 m × 0.25 mm, 0.25 μm; Restek); temperature program = start at 150 °C, hold for 2 min, increase at 15 °C/min to 320 °C, hold for 25 min; ion source and interface temperatures = 230 and 280 °C, respectively. Ions with *m/z* 361 and 217 were used for SIM. Data were processed by Labsolutions GCMSsolution software (version 4.20, Shimadzu), and calibration curves for absolute quantification were plotted in the range of 0.10–50.00 μg.

### 2.6. Extraction of Isoflavones and Their Analysis by UPLC-QTOF-MS

Isoflavone analysis was performed using a modification of a previously reported method [21]. Typically, a 100-mg aliquot of the lyophilized sample was extracted with 80 vol% aqueous ethanol (3 × 4 mL) in an ultrasonicator (3 × 15 min). The combined supernatants were filtered through 0.22-μm filters, diluted with methanol, and analyzed on a UPLC instrument (Waters, Milford, MA, USA) interfaced with a QTOF-MS spectrometer. An ACQUITY BEH C18 column (2.1 mm inner diameter × 100 mm length, 1.7 μm particle size) maintained at 35 °C and an injection volume of 2 μL were employed. The mobile phase corresponded to mixtures of 0.1 vol% aqueous formic acid (A) and 0.1 vol% formic acid in acetonitrile (B), and was supplied at 0.4 mL/min. The following gradient was used: 0 min, 90% A; 1 min, 90% A; 7 min, 85% A; 13 min, 70% A; 23 min, 20% A; 25 min, 0% A; 28 min, 0% A; and 30 min, 90% A. QTOF-MS analysis was performed in positive-ion mode over a range of *m/z* 100–1500 under the following conditions: capillary voltage = 2300 V; cone voltage = 50 V; ion source temperature = 110 °C; desolvation temperature = 450 °C; desolvation nitrogen gas flow rate = 800 L/h; and mass scan time = 0.25 s. Leucine enkephalin (*m/z* 556.2771) was used as a reference.

### 2.7. Statistical Analysis

Two differently fermented samples (AO or BS) were prepared for substrates extracted at three different temperatures (4, 25, or 55 °C). For metabolite analysis, three biological repetitions were used per sample. Relationships among or within group variations were probed by principal components analysis (PCA) and orthogonal partial least squares discriminant analysis (OPLS-DA) after unit variance (UV) scaling using soft independent modeling of class analogy (SIMCA) software (version 14.1, Umetrics, Sweden). To represent significantly different metabolites between two groups, the results of the Student’s *t*-test were presented as box-and-whisker plots created in MetaboAnalyst (version 4.0, http://www.metaboanalyst.ca/, McGill University, QC, Canada). Correlations among metabolites and between samples and metabolites were established by hierarchical cluster analysis (HCA) and heatmap visualization, respectively. Pearson’s correlation analysis was conducted using the statistical analysis system (SAS) software package (version 9.4, SAS Institute, Cary, NC, USA). In the case of heatmaps, the result was obtained from UV-scaled data. Both HCA and heatmap visualizations were performed using MultiExperiment Viewer (MeV) software (version 4.9.0, http://www.tm4.org/mev/, The Institute for Genomic Research, Rockville, MD, USA). For identified metabolite mappings, fold changes (FCs) were calculated for the average level of each metabolite as the BS fermented soybean/AO fermented soybean ratio. The outcome was transformed to log_2_ values (log_2_FC). PathVisio software (version 3.3.0, http://www.pathvisio.org, Maastricht University, Maastricht, The Netherlands) was employed to match log_2_FC values with the drawn metabolic pathways based on the *Arabidopsis thaliana* pathway in WikiPathways, *Glycine max* pathway in the KEGG database, and the published literature [22,23].

## 3. Results and Discussion

### 3.1. Metabolic Profiling and Multivariate Analysis of Fermented Soybeans

In general, primary metabolites such as amino acids, organic acids, sugars, and fatty acids are variously assembled as end-products of fermentations and interrelated with secondary metabolites as precursors [24]. Secondary metabolites, including isoflavones, tocopherols, and phytosterols, are also present in fermented soybeans and are important in view of their antioxidant, anti-inflammatory, and other activities [7,25]. Therefore, both primary and secondary metabolites were characterized in this study. GC-TOF-MS and GC-FID were respectively used to identify hydrophilic compounds (Figure S1, Table S4) and fatty acids (Figure S2, Table S5). GC–MS was used to analyze secondary lipophilic compounds (Figure S3, Table S6) and raffinose (Figure S4, Table S7), while UPLC-QTOF-MS was used to characterize isoflavones (Table S7). The obtained data were subjected to multivariate analysis to elucidate metabolic differences between the six types of samples (fermented by either AO or BS after water extraction at 4, 25, or 55 °C).

**Figure 1:**
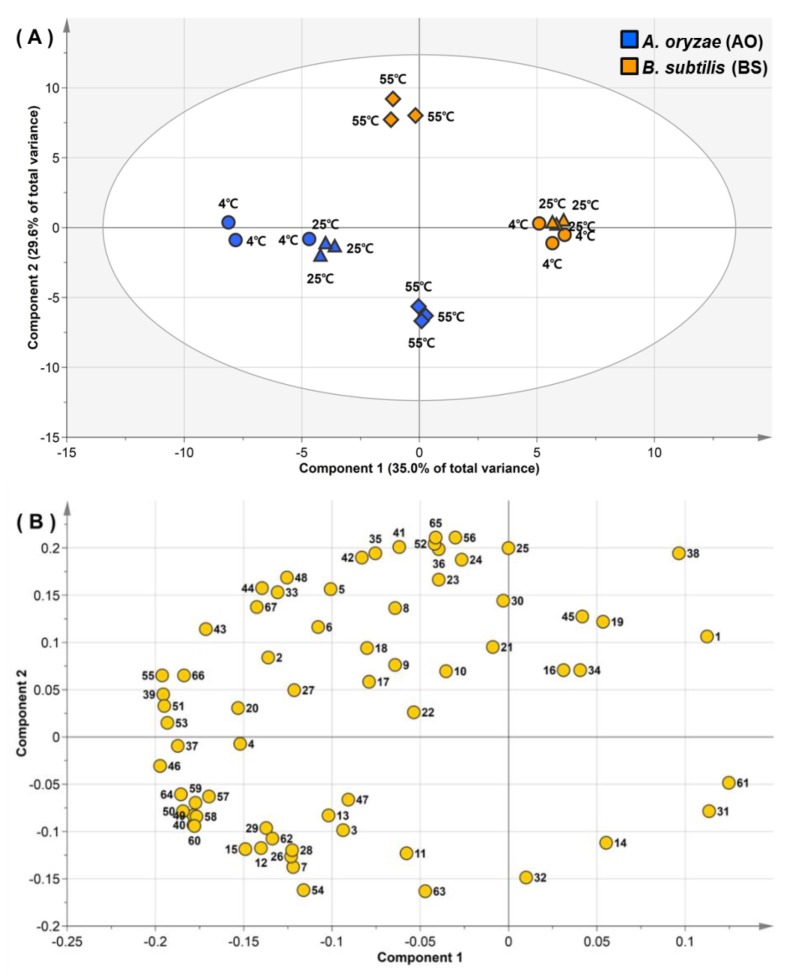
Score (**A**) and loading (**B**) plots of principal components 1 and 2 for the principal component analysis of hydrophilic metabolite, lipophilic metabolite, fatty acid, isoflavone, and raffinose data pertaining to *Aspergillus oryzae* (blue symbols) or *Bacillus subtilis* (yellow symbols) fermented soybeans after water extraction at 4 °C (circles), 25 °C (diamonds), and 55 °C (triangles). Plot annotation: 1, C14:0; 2, C16:0; 3, C16:1; 4, C18:0; 5, C18:1; 6, C18:2; 7, C18:3n6; 8, C18:3; 9, C20:0; 10, C20:3n6; 11, C20:5n3; 12, C22:0; 13, C22:1; 14, C22:5n3; 15, C22:6n3; 16, C24:0; 17, C20-ol; 18, C21-ol; 19, C22-ol; 20, C23-ol; 21, δ-tocopherol; 22, C26-ol; 23, β-tocopherol; 24, γ-tocopherol; 25, α-tocopherol; 26, campesterol; 27, C30-ol; 28, stigmasterol; 29, β-sitosterol; 30, β-amyrin; 31, raffinose; 32, lactic acid; 33, alanine; 34, glycolic acid; 35, valine; 36, urea; 37, serine; 38, ethanolamine; 39, phosphoric acid; 40, glycerol; 41, leucine; 42, isoleucine; 43, proline; 44, glycine; 45, succinic acid; 46, glyceric acid; 47, fumaric acid; 48, threonine; 49, β-alanine; 50, malic acid; 51, aspartic acid; 52, methionine; 53, pyroglutamic acid; 54, γ-aminobutyric acid; 55, glutamic acid; 56, phenylalanine; 57, asparagine; 58, glutamine; 59, citric acid; 60, fructose; 61, galactose; 62, glucose; 63, mannitol; 64, inositol; 65, tryptophan; 66, genistein; 67, daidzein.

Among the multivariate descriptions, PCA is well known as an unsupervised technique and the initial step of multivariate analysis used to outline overall data with dimension reduction [18]. Thus, data pertaining to 67 metabolites were initially subjected to PCA to identify the gross variability of all fermented samples (Figure 1). The two highest-rank principal components, accounting for 64.6% of the total variance, separated all fermented soybeans by inoculated microbe type. The first principal component (PC1) distinguished the fermented soybeans after extraction at 4 or 25 °C by AO and BS, accounting for 35.0% of the total variance. Among the 67 metabolites, 57 featured negative PC1 values, which implied that AO produced larger amounts of most metabolites than BS in soybeans extracted at 4 or 25 °C. The main metabolic loadings strongly correlated with the negative values of PC1 were glyceric acid, glutamic acid, phosphoric acid, aspartic acid, pyroglutamic acid, and serine. The second principal component (PC2), accounting for 29.6% of the total variance, separated soybeans extracted at 55 °C based on differences in fermenter species. In the PC2 loading, most amino acids and tocopherols had positive values and were clustered together, with the exception of γ-aminobutyric acid (GABA), glutamine, β-alanine, and asparagine. The representative metabolites for the positive values of PC2 were phenylalanine, tryptophan, α-tocopherol, and γ-tocopherol. On the other hand, sugar alcohols, organic acids, phytosterols, and sugars were grouped with negative values of PC2. The metabolites mainly attributable to the negative values of PC2 were mannitol, lactic acid, campesterol, and glucose. PC2 loading plots indicated that BS produced comparatively higher levels of numerous amino acids and tocopherols for soybeans extracted at 55 °C, whereas AO produced abundant sugar alcohols, organic acids, phytosterols, and sugars under identical conditions.

Ultimately, PCA results demonstrated that soybean metabolic changes were mainly attributable to fermentation by AO and BS. For the same microbe species, soybeans extracted at 55 °C were notably different from those extracted at 4 or 25 °C, which suggested that the metabolic strategies of both AO and BS at 55 °C were different from those at 4 or 25 °C.

**Figure 2:**
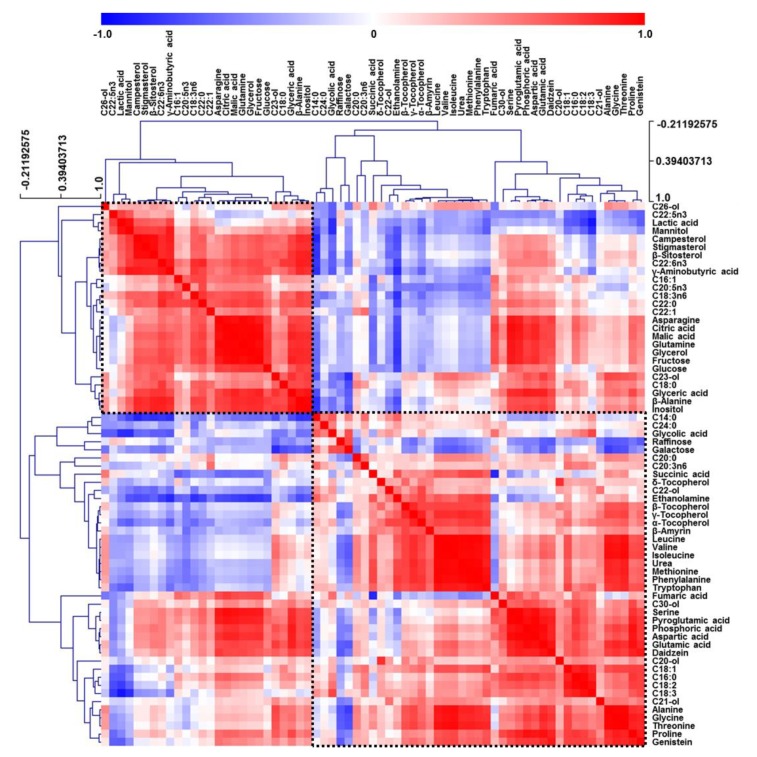
Correlation matrix obtained for hydrophilic metabolites, lipophilic metabolites, fatty acids, isoflavones, and raffinose extracted from soybeans. Each square indicates the Pearson’s correlation coefficient of a pair of compounds, with the value of this coefficient represented by the intensity of blue or red colors, as indicated on the color scale.

Correlation analysis relates the results of quantitative metabolite profiling with clustering coefficients [26]. As metabolite correlations were mainly represented as a result of highly significant differences between samples, a comparison of metabolite properties had to be introduced into the correlation matrix to account for unexpected assortative features. Therefore, HCA was performed to examine correlations between the 67 metabolites in all fermented soybean samples (Figure 2). For pairwise correlational comparison, red and blue color scales were viewed as contrasts of 1 and −1, respectively.

All metabolites were largely grouped into two correlations, and some metabolites (e.g., tocopherols, phytosterols, and organic acids) produced by closely related pathways were clustered together. In addition, HCA patterns resembled those of PCA in Figure 1, e.g., amino acids and tocopherols, which had positive values of PC2 in PCA loading plots, were positively correlated together in one group, while sugar alcohols, organic acids, phytosterols, and sugars, which had negative values of PC2 in the loading plots, were positively clustered in the other group. Interestingly, GABA, glutamine, β-alanine, and asparagine were closely correlated within the sugar alcohol, organic acid, phytosterol, and sugar group rather than within the amino acid and tocopherol group. Likewise, HCA results revealed that most highly correlated compounds were clustered on the basis of metabolic differences of soybean samples and related biological pathways.

### 3.2. Comparison of Phytochemical Profiles of AO-/BS-Fermented Soybeans Extracted at 4, 25, or 55 °C

**Figure 3:**
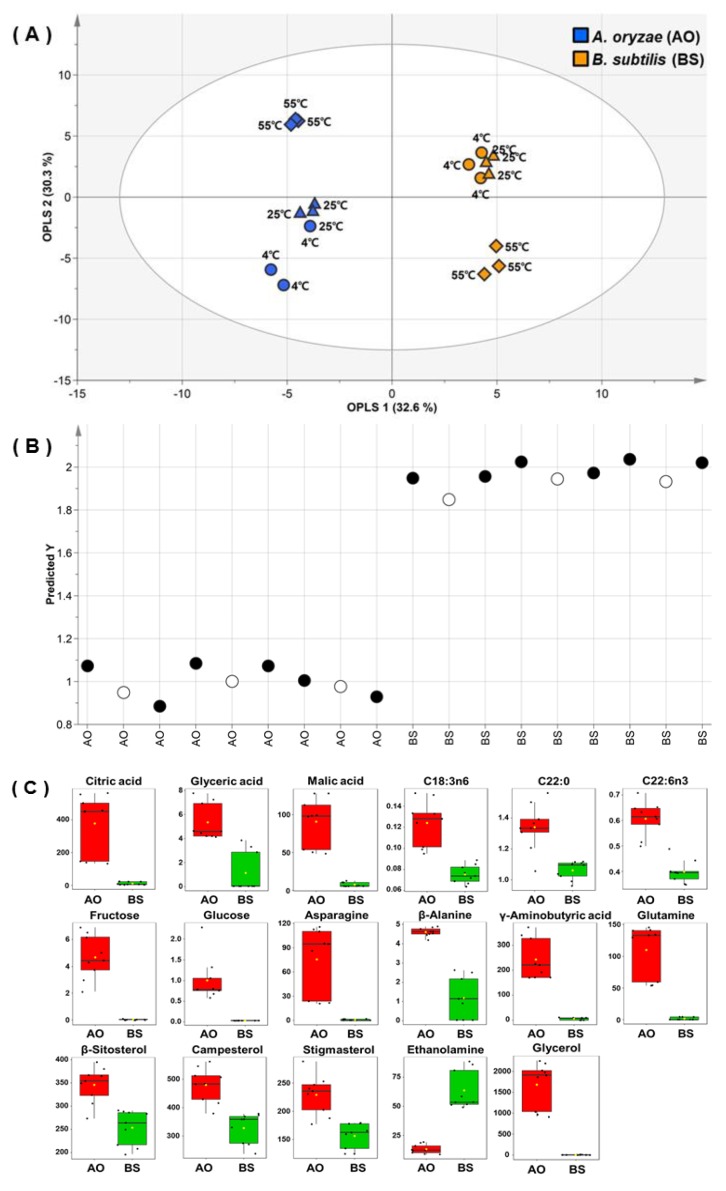
Orthogonal partial least squares discriminant analysis (OPLS-DA) score (**A**) and external validation test (**B**) plots for hydrophilic metabolite, lipophilic metabolite, fatty acid, isoflavone, and raffinose data obtained for soybeans fermented by *Aspergillus oryzae* (AO, blue symbols) or *Bacillus subtilis* (BS, yellow symbols) after water extraction at 4 °C (circles), 25 °C (diamonds), or 55 °C (triangles). (**C**) Box plots of metabolites significantly different between AO- and BS-fermented soybeans. On the basis of variable importance in the projection >1.0 in the OPLS-DA model and the *p* < 0.0001 in the *t*-test for all metabolites, 17 metabolites were selected.

Although unsupervised analyses such as PCA and HCA are useful for investigating overall sample patterns, they do not allow one to easily identify common properties for each variation. On the other hand, OPLS-DA is a suitable tool for reducing non-correlated variation and model complexity with improved interpretational ability, and its appropriate application can yield beneficial insights into common and predictive features in each sample group [27]. As metabolic differences were mainly observed for different fermenters in PCA results, OPLS-DA was used to identify the separations of all fermented soybean samples by inoculated microbes without considering substrate extraction temperatures (Figure 3A). The quality of the OPLS-DA model was represented by fit goodness (*R*^2^). The quality parameter of *X* variables (*R*_X_^2^ = 0.629) was measured as 32.6% and 30.3% of the total variance for the first and second principal components, respectively. The prediction accuracy of the OPLS-DA model was represented by prediction goodness (*Q*^2^), which was estimated as 0.975 and thus indicated excellent prediction power (*Q*^2^ > 0.9) [28]. Thus, the OPLS-DA result confirmed that the metabolite contents of AO- and BS-fermented soybeans were significantly different.

External validation was performed to more accurately evaluate the prediction ability of the model based on different sample sets (Figure 3B). One third of the samples from data sets were retained as test data, and OPLS-DA was performed for the training set. *R*_X_^2^ and *Q*^2^ were obtained as 0.595 and 0.963, respectively. In addition to the cross-validated *Q*^2^ value, the prediction strength was estimated based on the root mean square error of prediction (RMSEP), the low values of which indicate good prediction ability [28]. The created model had an RMSEP of 0.0752, which indicated good predictive power. Variable importance in the projection (VIP) was used to identify metabolites most significantly contributing to variation (Figure S5). The model influential variation featured a VIP value of >1.0. With *p* < 0.0001, 17 variables were considered as metabolites distinguishing between AO- and BS-fermented soybeans (Figure 3C). These notable metabolites, i.e., organic acids (glyceric acid, malic acid, and citric acid) and sugars (glucose and fructose), were more abundant in AO-fermented soybeans. This result was similar to that of a previous study, which revealed that the influence of sugars on organic acid production resulted in lower pH during fermentation by *Aspergillus niger* [29]. In addition, some researchers have reported that *Aspergillus* fungi tolerate acidic conditions that otherwise limit normal microbial growth [30,31]. Therefore, our results revealed that the elevated sugar and organic acid levels in AO-fermented soybeans were related to the ability of AO to survive under acidic conditions. On the other hand, as *Bacillus* bacteria can survive only at neutral pH [32], we expected BS-induced fermentation to result in a decreased production of organic acids. In summary, AO activated carbohydrate metabolism in soybeans better than BS, as the former microbes featured a broader range of survival conditions than the latter.

**Figure 4:**
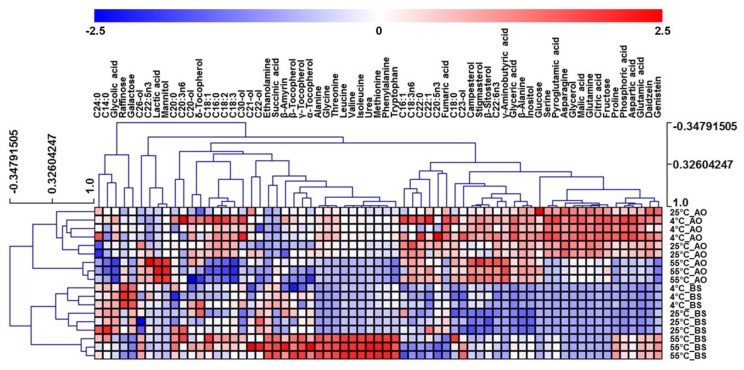
Heatmap representing differences in the relative metabolite concentrations of soybeans fermented by *Aspergillus oryzae* (AO) or *Bacillus subtilis* (BS). Increased and decreased contents of metabolites are shown by red and blue colors, respectively.

Although OPLS-DA helped to identify compounds important for dividing the two types of fermented soybeans, it was of limited use for distinguishing the metabolite fluctuations observed for different extraction temperatures within each of the two groups. Thus, heatmap visualization was performed to compare the relative metabolic changes of soybeans for different extraction temperatures and fermenters (Figure 4). As a result, two clusters with distinct microbe treatment patterns were identified. Within each cluster, soybeans extracted at 55 °C were different from those extracted at 4 or 25 °C.

In AO-fermented soybean groups, soybeans extracted at 55 °C showed decreased levels of fatty acids (C16:0, C18:1, C18:2, and C18:3) and increased levels of lactic acid. Although fatty acid release commonly takes place under the action of microbial lipases, the preference of lipase activity depends on the initial substrates [16,33]. As regards substrates extracted at different temperatures, a previous study showed that high storage temperatures facilitate the oxidation of soybean fatty acids, resulting in their decreased contents [34]. Particularly, it has been shown that unsaturated fatty acids are sensitive to oxidation and have higher levels of peroxide than saturated fatty acids at warm temperatures [35,36,37]. Our results suggested that some fatty acids in soybean residues could be decreased and oxidized rapidly under 55 °C extraction conditions. Interestingly, in low-fat substrates, AO changes its metabolic preference from fatty acid and lipase production to lactic acid production [17]. Thus, the decreased fatty acid levels of soybeans extracted at 55 °C may contribute to the modification of fermentative strategies, increasing lactic acid production during AO fermentation.

For the BS-fermented group, extraction at 55 °C resulted in increased levels of amino acids (alanine, glycine, threonine, leucine, valine, isoleucine, methionine, phenylalanine, tryptophan, proline, and aspartic acid) and tocopherols (α-tocopherol, β-tocopherol, and γ-tocopherol). The observed differences of amino acid contents were attributed to the intensified combination effects of heat treatment and BS fermentation. Soybean heat treatment has been demonstrated to induce the breakdown of protein peptide bonds and the release of amino acids [16]. Moreover, previous researches also showed that the increased solubility of amino acids in the medium leads to additional acceleration of BS-induced fermentation and high protease activity [8,38]. Hence, the observed results suggest that proteases contained in BS robustly accelerated amino acid release because of the enhanced breakdown of proteins to amino acids in warm water.

### 3.3. Metabolic Disparity and Pathway Analysis of Soybeans Fermented by AO or BS after Extraction at 55 °C

**Figure 5:**
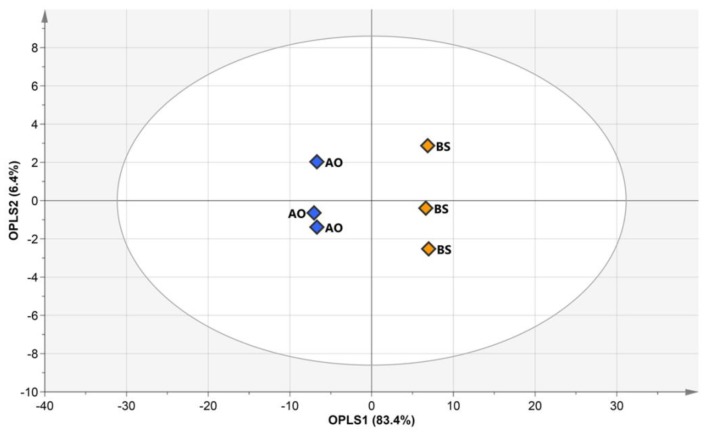
Orthogonal partial least squares discriminant analysis score plots for hydrophilic metabolite, lipophilic metabolite, fatty acid, isoflavone, and raffinose data of soybeans fermented by *Aspergillus oryzae* (AO, blue symbols) or *Bacillus subtilis* (BS, yellow symbols) after water extraction at 55 °C.

Although the metabolites of fermented soybeans were mainly influenced by the inoculated microbe type, they were also affected by extraction temperature in each fermenter group. Thus, the effects of AO and BS had to be compared under identical conditions. A temperature of 55 °C was chosen for comparison because of the rapid elevation of metabolic changes under these conditions. Thus, AO- or BS-fermented soybeans after extraction at 55 °C were subjected to OPLS-DA to exactly investigate the differences between soybean metabolites (Figure 5). The corresponding *R*_X_^2^ and *Q*^2^ values were obtained as 0.897 and 0.996, respectively, and were indicative of excellent predictive power. OPLS 1 and OPLS 2 accounted for 83.4% and 6.4% of the total variance, respectively. VIP scores were used to determine metabolites mainly contributing to variation (Figure S6). Among the 67 metabolites, 26 variables selected based on VIP > 1 and *p* < 0.0001 contributed to significant differences and played the greatest roles in discriminating between the two types of soybeans (Figure S7). In AO-fermented soybeans, three amino acids (asparagine, GABA, and glutamine), three organic acids (citric acid, lactic acid, and malic acid), and two sugar alcohols (glycerol and mannitol) were abundant, whereas BS-fermented soybeans were characterized by elevated levels of 13 amino acids (alanine, aspartic acid, glutamic acid glycine, isoleucine, leucine, methionine, phenylalanine, proline, serine, threonine, tryptophan, and valine), one organic acid (urea), two isoflavones (daidzein, genistein), and two other compounds (phosphoric acid and ethanolamine). *t*-Test results were used to directly identify the different metabolites mediated by the two types of samples. However, these chemometric approaches alone might be insufficient for interpreting the causes of the differences related to metabolic pathways [39].

**Figure 6:**
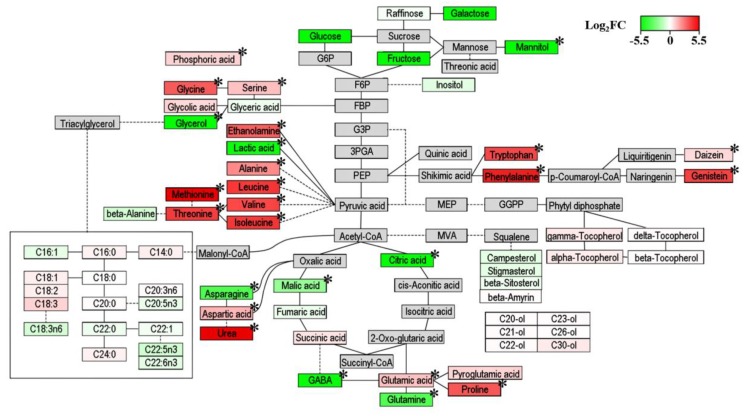
Metabolic pathway visualization and relative metabolite abundance for soybeans fermented by *Aspergillus oryzae* (AO) or *Bacillus subtilis* (BS) after water extraction at 55 °C. Fold changes from AO to BS were converted into log_2_FC (BS/AO) values ranging from −5.50 to 5.50. Positive log_2_FC values imply higher levels in BS-fermented soybeans and are shown in red, while negative log_2_FC values imply higher levels in AO-fermented soybeans and are shown in green. Extremely significant differences between AO and BS groups were identified by the *t*-test (* *p* < 0.0001).

Pathway analysis is a powerful way of gaining insights into the metabolic change patterns associated with two different distributers. Based on all of the metabolites of soybean substrates, inoculated microbes produced or decomposed metabolites in some parts of the metabolic pathway of soybeans during fermentation [22]. However, the complex changes in metabolites according to different fermenters can be schemed in the metabolic pathway of their substrates [15,22]. To correlate the relative metabolite contents of AO- and BS-fermented soybeans with the related biosynthetic pathways, log_2_FC data were visualized by PathVisio (Figure 6). For each metabolite, green-to-red gradient colors show log_2_FC values from −5.5 to 5.5 and indicate the relative abundance of metabolites. Shades of green (−5.5 < log_2_FC < 0) refer to higher levels in AO-fermented soybeans, while shades of red (0 < log_2_FC < 5.5) imply higher levels in BS-fermented soybeans. In addition, metabolites significantly different (VIP > 1, *p* < 0.0001) between the two experimental conditions in OPLS-DA results are indicated by asterisks (*).

Due to carbohydrate metabolism, most sugars, sugar alcohols, and organic acids (especially glycerol, mannitol, citric acid, malic acid, and lactic acid) were more abundant in AO-fermented soybeans. Three sugars (galactose, glucose, and fructose) and two sugar alcohols (glycerol and mannitol) were only observed in AO-fermented soybeans. The abundances of sugars, sugar alcohols, and organic acids in AO-fermented samples were similar to those found in previous studies [29,40]. Considering sugar metabolism, AO was shown to have a higher activity of α-amylase, which converts polysaccharides into monosaccharides. Furthermore, increased sugar reduction activates glycolysis and the TCA cycle in carbohydrate metabolism. In the late stage of fermentation, sugar alcohols are accumulated because of citric acid fermentation [41]. Thus, for soybeans extracted at high temperature, AO fermentation was concluded to afford larger amounts of sugars, sugar alcohols, and organic acids than BS fermentation because of the accelerated energy metabolism in the former case.

Regarding amino acid metabolism, the levels of most amino acids (including glycine, serine, alanine, leucine, valine, isoleucine, methionine, threonine, aspartic acid, glutamic acid, proline, tryptophan, and phenylalanine) were maintained at much higher values in the case of BS fermentation. Amino acids are nitrogen sources for both starting fermentation and growing microorganisms in general [42]. In a previous study, the accumulation of amino acids and protease activity were more pronounced in *Bacillus* than in *Aspergillus* [1,40]. Thus, based on our results, we anticipated that increased protease activity might increase the levels of amino acids in soybeans fermented by BS. Regarding biosynthetic pathways, this result was related to the low contents of organic acids, which are amino acid precursors, in BS-fermented soybeans [43]. Previous works showed that amino acids such as isoleucine, valine, and methionine are synthesized from TCA cycle intermediates during fermentation by *Bacillus* [44,45]. Thus, our results implied that protease activity and the degree of organic acid to amino acid conversion were greater for BS than for AO in the case of warm water-extracted soybeans. In addition, fermentation by BS released essential and nonessential amino acids, which allowed the thus processed soybeans to be regarded as a functional food capable of providing appropriate quantities of all amino acids to maintain the normal growth and physiological functions of immune and central nervous systems in humans and animals [46].

AO-fermented soybeans featured elevated levels of GABA, glutamine, and asparagine, which were ascribed to the high levels of glutamate decarboxylase (GAD, produces GABA from glutamic acid) [40,47] and glutamine synthetase (GS, accounts for the biosynthesis of glutamine) [48] in AO. In agreement with previous results, we demonstrated a significant increase of GABA and glutamine levels and a decrease of glutamic acid levels in AO-fermented soybeans. The enhanced levels of GABA and glutamine in AO-fermented soybeans also allowed them to be viewed as a functional food. In view of the fact that GABA acts as an inhibitory neurotransmitter in human and animal brains [49], its intake can prevent the development of Alzheimer’s disease, epilepsy, and sleep disorders as well as have hypotensive, tranquilizing, diuretic, and antidiabetic effects. A well-balanced diet should also contain glutamine, a well-known essential amino acid required to maintain and promote the proliferation of human skeletal muscle cells [50].

As regards secondary metabolism, we observed that two isoflavones (daidzein and genistein) were relatively abundant in BS groups. Isoflavones, known as phytoestrogens, exist in soybeans in two chemical forms, namely as aglycones and glucosides. Although the latter form is predominant, food processing methods such as heating and fermentation convert isoflavone glucosides into their aglycone forms by activating β-glucosidase [51]. In particular, β-glucosidase activity was shown to be higher in *Koji* rice fermented by *Bacillus* than in that fermented by *Aspergillus* [40]. Therefore, our results implied that the abundance of isoflavone aglycones in BS-fermented soybeans was related to increased β-glucosidase activity. From a nutritional point of view, the high contents of daidzein and genistein in BS-fermented soybeans were beneficial to human health. Past studies revealed that in order for isoflavones to be absorbed by passive transport, their β-glucosidic linkages need to be hydrolyzed by bacteria in the intestine [52,53]. Hence, isoflavone aglycones are more bioavailable than their glucoside forms and thus exhibit beneficial effects, e.g., prevent the development of cardiovascular diseases and increase bone density [54].

## 4. Conclusions

This study evaluated the metabolic differences between AO- and BS-fermented soybeans extracted at 4, 25, or 55 °C. Overall, soybean samples were grouped on the basis of fermenter type, which suggested that this type was the main factor responsible for the above differences. Interestingly, substrate extraction temperature also influenced the metabolic changes of fermented soybeans within the two fermenter groups, that is, soybeans extracted at 55 °C were different from those extracted at 4 or 25 °C. Within the AO group, soybeans extracted at 55 °C showed comparatively high amounts of lactic acid and reduced levels of fatty acids. In the BS group, elevated levels of amino acids were observed in soybeans extracted at 55 °C. These results implied that soybean extraction at 55 °C contributed to the activated metabolic changes of fermented products. For further investigation of differences between AO and BS, soybeans extracted at 55 °C were selected as a uniform substrate. Contrasting metabolic changes for AO and BS were explained by considering metabolic pathways. AO-fermented soybeans featured higher levels of sugar alcohols and organic acids, which was indicative of energy metabolism, and were also rich in GABA and glutamine because of the increased GAD and GS activities. On the other hand, the levels of most essential and nonessential amino acids were higher in BS-fermented soybeans, which were ascribed to elevated protease activities and the usage of organic acids as precursors. BS fermentation also resulted in comparatively high amounts of isoflavone aglycones. Hence, our results showed that different metabolic strategies of AO and BS as well as substrate conditions were largely responsible for the metabolic profile differences of fermented soybeans. The obtained insights are expected to deepen our understanding of variously fermented foods and help to optimize the processing steps for selectively enhancing the contents of the desired nutrients.

## Supplementary Materials

The following are available online at https://www.mdpi.com/2304-8158/9/2/117/s1: Figure S1: GC-TOF-MS chromatogram of *A. oryzae* (AO) fermented soybeans after extraction at 4 °C. 1, Lactic acid; 2, Alanine; 3, Glycolic acid; 4, Valine; 5, Urea; 6. Serine-1; 7, Ethanolamine; 8, Phosphoric acid; 9, Glycerol; 10, Leucine; 11, Isoleucine; 12, Proline; 13, Glycine; 14, Succinic acid; 15, Glyceric acid; 16, Fumaric acid; 17, Serine-2; 18, Threonine; 19, β-Alanine; 20, Malic acid; 21, Aspartic acid; 22, Methionine; 23, Pyroglutamic acid; 24, γ-Aminobutyric acid; 25, Glutamic acid; 26, Phenylalanine; 27, Asparagine; 28, Ribitol (internal standard); 29, Glutamine; 30, Citric acid; 31, Fructose-1; 32, Fructose-2; 33, Galactose; 34, Glucose; 35, Mannitol; 36, Inositol; 37, Tryptophan, Figure S2: GC-FID chromatogram of *A. oryzae* (AO) fermented soybeans after extraction at 4 °C. 1, C14:0; 2, Pentadecanoic acid (internal standard); 3, C16:0; 4, C16:1; 5, C18:0; 6, C18:1; 7, C18:2; 8, C18:3n6; 9, C18:3; 10, C20:0; 11, C20:3n6; 12, C20:5n3; 13, C22:0; 14, C22:1; 15, C22:5n3; 16, C22:6n3; 17, C24:0, Figure S3: EIC (A) and selected ion monitoring (SIM) chromatogram (B) of secondary lipophilic compounds as TMS derivatives. GC–MS was used to analyze lipophilic metabolites of *A. oryzae* (AO) fermented soybeans after extraction at 4 °C. The selected compounds are displayed in (B). Inverted triangles represent peak of target compound. 1, C20-ol; 2, C21-ol; 3, C22-ol; 4, C23-ol; 5, 5α-Cholestane (internal standard); 6, δ-Tocopherol; 7, C26-ol; 8, β-Tocopherol; 9, γ-Tocopherol; 10, α-Tocopherol; 11, Campesterol; 12, C30-ol; 13, Stigmasterol; 14, β-Sitosterol; 15, β-Amyrin, Figure S4: Representative selected ion monitoring (SIM) chromatogram of ribitol (internal standard) and raffinose in *A. oryzae* (AO) fermented soybeans after extraction at 4 °C, Figure S5: The influence of variables used to separate the extracted and then fermented soybeans by two types of microbes. Plot annotation 1, Ethanolamine; 2, γ-Aminobutyric acid; 3, Glycerol; 4, Fructose; 5, β-Alanine; 6, Malic acid; 7, C22:6n3; 8, Glutamine; 9, Inositol; 10, Citric acid; 11, Glyceric acid; 12, C18:3n6; 13, C22:0; 14, Campesterol; 15, Stigmasterol; 16, Asparagine; 17, β-Sitosterol; 18, Glucose; 19, Serine; 20, Pyroglutamic acid; 21, C14:0; 22, Mannitol; 23, Aspartic acid; 24, Phosphoric acid; 25, Glutamic acid; 26, α-Tocopherol; 27, Tryptophan; 28, Daidzein; 29, Phenylalanine; 30, C18:0; 31, Proline; 32, C16:1; 33, C22:1; 34, Methionine; 35, Lactic acid; 36, C22-ol; 37, C23-ol; 38, Urea; 39, Leucine; 40, γ-Tocopherol; 41, C20:5n3; 42, C16:0; 43, Genistein; 44, C18:1; 45, C18:3; 46, Valine; 47, Glycine; 48, C18:2; 49, Isoleucine; 50, Threonine; 51, Fumaric acid; 52, Succinic acid; 53, C30-ol; 54, Alanine; 55, β-Tocopherol; 56, β-Amyrin; 57, Glycolic acid; 58, C22:5n3; 59, Galactose; 60, C24:0; 61, Raffinose; 62, δ-Tocopherol; 63, C20-ol; 64, C20:3n6; 65, C21-ol; 66, C20:0; 67, C26-ol, Figure S6: The influence of variables used to separate the soybeans extracted at 55 °C by two types of microorganisms. Plot annotation 1, Genistein; 2, Mannitol; 3, Daidzein; 4, Citric acid; 5, Methionine; 6, Glutamine; 7, Valine; 8, Tryptophan; 9, Glycerol; 10, Leucine; 11, Phenylalanine; 12, Isoleucine; 13, Proline; 14, Threonine; 15, Glycine; 16, Lactic acid; 17, γ-Aminobutyric acid; 18, Urea; 19, Asparagine; 20, Ethanolamine; 21, Alanine; 22, Malic acid; 23, Aspartic acid; 24, Serine; 25, Glutamic acid; 26, Phosphoric acid; 27, Glycolic acid; 28, β-Alanine; 29, β-Sitosterol; 30, Campesterol; 31, C18:3; 32, C18:3n6; 33, Succinic acid; 34, Pyroglutamic acid; 35, Glucose; 36, γ-Tocopherol; 37, Inositol; 38, Galactose; 39, Fructose; 40, Fumaric acid; 41, Stigmasterol; 42, C22:0; 43, C22:6n3; 44, α-Tocopherol; 45, β-Tocopherol; 46, C18:2; 47, C18:1; 48, C20:5n3; 49, C24:0; 50, C14:0; 51, Raffinose; 52, C16:0; 53, β-Amyrin; 54, Glyceric acid; 55, C30-ol; 56, C22:1; 57, C20:0; 58, C22-ol; 59, C22:5n3; 60, C26-ol; 61, δ-Tocopherol; 62, C16:1; 63, C21-ol; 64, C20:3n6; 65, C20-ol; 66, C23-ol; 67, C18:0, Figure S7: Box plots of significantly different metabolites between *A. oryzae* (AO) and *B. subtilis* (BS) fermented soybeans after extraction at 55 °C. On the basis of variable importance in the projection (VIP) value of > 1.0 in the OPLS-DA model and the *p*-value (*p* < 0.0001) in the *t*-test for all metabolites, 26 metabolites were selected, Table S1: Relative retention times (RRT) and mass spectrometry data of identified hydrophilic compounds with GC-TOF-MS, Table S2: Relative retention times (RRT) of fatty acid methyl esters (FAME) mixture and other fatty acids for quantification with GC-FID, Table S3: Relative retention times (RRT) and mass spectrometry data of secondary lipophilic compounds with GC–MS, Table S4: Composition and content (ratio/g) of hydrophilic compounds in soybeans with GC-TOF-MS analysis, Table S5: Composition and content (μg/mg) of fatty acids in soybeans with GC-FID analysis, Table S6: Composition and content (μg/g) of secondary lipophilic compounds in soybeans with GC–MS analysis, Table S7: Composition and content of raffinose (μg/mg) and isoflavones (μg/mL) with GC–MS and UPLC-QTOF-MS analysis.

## References

[1] Teng D., Gao M., Yang Y., Liu B., Tian Z., Wang J. (2012). Bio-modification of soybean meal with *Bacillus subtilis* or *Aspergillus oryzae*. Biocatal. Agric. Biotechnol..

[2] Sharma S., Goyal R., Barwal S. (2013). Domestic processing effects on physicochemical, nutritional and anti-nutritional attributes in soybean (*Glycine max* L. Merill). Int. Food Res. J..

[3] Rickert D.A., Meyer M.A., Hu J., Murphy P.A. (2004). Effect of extraction pH and temperature on isoflavone and saponin partitioning and profile during soy protein isolate production. J. Food Sci..

[4] Palavalli M.H., Natarajan S.S., Wang T.T., Krishnan H.B. (2012). Imbibition of soybean seeds in warm water results in the release of copious amounts of Bowman–Birk protease inhibitor, a putative anticarcinogenic agent. J. Agric. Food Chem..

[5] Le X.T., Vi L., Luu V.L.L., Toan T.Q., Bach L.G., Truc T.T., Ha P.T.H. (2019). Extraction process of polyphenols from soybean (*Glycine Max* L.) sprouts: Optimization and evaluation of antioxidant activity. Processes.

[6] Calderón de la Barca A.M., Ruiz-Salazar R., Jara-Marini M.E. (2000). Enzymatic hydrolysis and synthesis of soy protein to improve its amino acid composition and functional properties. J. Food Sci..

[7] El-Adawy T.A. (2002). Nutritional composition and antinutritional factors of chickpeas (*Cicer arietinum* L.) undergoing different cooking methods and germination. Plant Foods Hum. Nutr..

[8] Hur S.J., Lee S.Y., Kim Y.C., Choi I., Kim G.B. (2014). Effect of fermentation on the antioxidant activity in plant-based foods. Food Chem..

[9] Shin D., Jeong D. (2015). Korean traditional fermented soybean products: *Jang*. J. Ethn. Foods.

[10] Barbesgaard P., Heldt-Hansen H.P., Diderichsen B. (1992). On the safety of *Aspergillus oryzae*: A review. Appl. Microbiol. Biotechnol..

[11] Vishwanatha K.S., Rao A.A., Singh S.A. (2010). Acid protease production by solid-state fermentation using *Aspergillus oryzae* MTCC 5341: Optimization of process parameters. J. Ind. Microbiol. Biotechnol..

[12] Shrestha A.K., Dahal N.R., Ndungutse V. (2010). *Bacillus* fermentation of soybean: A review. J. Food Sci. Technol. Nepal.

[13] Mozzi F., Ortiz M.E., Bleckwedel J., De Vuyst L., Pescuma M. (2013). Metabolomics as a tool for the comprehensive understanding of fermented and functional foods with lactic acid bacteria. Food Res. Int..

[14] Hong K.J., Lee C.H., Kim S.W. (2004). *Aspergillus oryzae* GB-107 fermentation improves nutritional quality of food soybeans and feed soybean meals. J. Med. Food.

[15] Seo H.S., Lee S., Singh D., Shin H.W., Cho S.A., Lee C.H. (2018). Untargeted metabolite profiling for *koji*-fermentative bioprocess unravels the effects of varying substrate types and microbial inocula. Food Chem..

[16] Trugo L.C., Donangelo C.M., Trugo N.M.F., Bach Knudsen K.E. (2000). Effect of heat treatment on nutritional quality of germinated legume seeds. J. Agric. Food Chem..

[17] Mahboubi A., Ferreira J., Taherzadeh M., Lennartsson P. (2017). Production of fungal biomass for feed, fatty Acids, and glycerol by *Aspergillus oryzae* from fat-rich dairy substrates. Fermentation.

[18] Park S.Y., Lim S.H., Ha S.H., Yeo Y., Park W.T., Kwon D.Y., Park S.U., Kim J.K. (2013). Metabolite profiling approach reveals the interface of primary and secondary metabolism in colored cauliflowers (*Brassica oleracea* L. ssp. botrytis). J. Agric. Food Chem..

[19] Kim M.S., Baek S.A., Park S.Y., Baek S.H., Lee S.M., Ha S.H., Lee Y.T., Choi J., Im K.H., Kim J.K. (2016). Comparison of the grain composition in resveratrol-enriched and glufosinate-tolerant rice (*Oryza sativa*) to conventional rice using univariate and multivariate analysis. J. Food Compos. Anal..

[20] Kim T.J., Lee K.B., Baek S.A., Choi J., Ha S.H., Lim S.H., Park S.Y., Yeo Y., Park S.U., Kim J.K. (2015). Determination of lipophilic metabolites for species discrimination and quality assessment of nine leafy vegetables. J. Korean Soc. Appl. Biol. Chem..

[21] Lee J.H., Baek I.Y., Choung M.G., Ha T.J., Han W.Y., Cho K.M., Ko J.M., Jeong S.H., Oh K.W., Park K.Y. (2008). Phytochemical constituents from the leaves of soybean [*Glycine max* (L.) Merr.]. Food Sci. Biotechnol..

[22] Kim J., Choi J.N., Maria John K.M., Kusano M., Oikawa A., Saito K., Lee C.H. (2012). GC–TOF-MS-and CE–TOF-MS-based metabolic profiling of cheonggukjang (fast-fermented bean paste) during fermentation and its correlation with metabolic pathways. J. Agric. Food Chem..

[23] Lee S.Y., Lee S., Lee S., Oh J.Y., Jeon E.J., Ryu H.S., Lee C.H. (2014). Primary and secondary metabolite profiling of *doenjang*, a fermented soybean paste during industrial processing. Food Chem..

[24] Drew S.W., Demain A.L. (1977). Effect of primary metabolites on secondary metabolism. Annu. Rev. Microbiol..

[25] Shi H., Nam P.K., Ma Y. (2010). Comprehensive profiling of isoflavones, phytosterols, tocopherols, minerals, crude protein, lipid, and sugar during soybean (*Glycine max*) germination. J. Agric. Food Chem..

[26] Steuer R. (2006). On the analysis and interpretation of correlations in metabolomic data. Brief. Bioinform..

[27] Worley B., Powers R. (2016). PCA as a practical indicator of OPLS-DA model reliability. Curr. Metab..

[28] Eriksson L., Byrne T., Johansson E., Trygg J., Vikström C. (2013). Appendix II: Statistics. Multi-and Megavariate Data Analysis Basic Principles and Applications.

[29] Magnuson J.K., Lasure L.L., Tkacz J.S., Lange L. (2004). Organic acid production by filamentous fungi. Advances in Fungal Biotechnology for Industry, Agriculture, and Medicine.

[30] Carlsen M., Spohr A.B., Nielsen J., Villadsen J. (1996). Morphology and physiology of an α-amylase producing strain of *Aspergillus oryzae* during batch cultivations. Biotechnol. Bioeng..

[31] Chipeta Z.A., Du Preez J.C., Christopher L. (2008). Effect of cultivation pH and agitation rate on growth and xylanase production by *Aspergillus oryzae* in spent sulphite liquor. J. Ind. Microbiol. Biotechnol..

[32] Liu H., Wang J., Liu X., Fu B., Chen J., Yu H.Q. (2012). Acidogenic fermentation of proteinaceous sewage sludge: Effect of pH. Water Res..

[33] Salihu A., Alam M.Z., AbdulKarim M.I., Salleh H.M. (2012). Lipase production: An insight in the utilization of renewable agricultural residues. Resour. Conserv. Recycl..

[34] Prabakaran M., Lee K.J., An Y., Kwon C., Kim S., Yang Y., Ahmad A., Kim S.H., Chung I.M. (2018). Changes in soybean (*Glycine max* L.) flour fatty-acid content based on storage temperature and duration. Molecules.

[35] Stewart O.J., Raghavan G.S.V., Orsat V., Golden K.D. (2003). The effect of drying on unsaturated fatty acids and trypsin inhibitor activity in soybean. Process Biochem..

[36] Mistry B.S., Min D.B. (1987). Effects of fatty acids on the oxidative stability of soybean oil. J. Food Sci..

[37] Colakoglu A.S. (2007). Oxidation kinetics of soybean oil in the presence of monoolein, stearic acid and iron. Food Chem..

[38] Zhang C., Wu D., Qiu X. (2018). Stimulatory effects of amino acids on γ-polyglutamic acid production by *Bacillus subtilis*. Sci. Rep..

[39] Rosato A., Tenori L., Cascante M., Carulla P.R.D.A., dos Santos V.A.M., Saccenti E. (2018). From correlation to causation: Analysis of metabolomics data using systems biology approaches. Metabolomics.

[40] Lee D., Lee S., Jang E., Shin H., Moon B., Lee C. (2016). Metabolomic profiles of *Aspergillus oryzae* and *Bacillus amyloliquefaciens* during rice *koji* fermentation. Molecules.

[41] Röhr M., Kubicek C.P., Zehentgruber O., Orthofer R. (1987). Accumulation and partial re-consumption of polyols during citric acid fermentation by *Aspergillus niger*. Appl. Microbiol. Biotechnol..

[42] Ko B.K., Ahn H.J., van den Berg F., Lee C.H., Hong Y.S. (2009). Metabolomic insight into soy sauce through ^1^H NMR spectroscopy. J. Agric. Food Chem..

[43] Kusano M., Fukushima A., Redestig H., Saito K. (2011). Metabolomic approaches toward understanding nitrogen metabolism in plants. J. Exp. Bot..

[44] Schilling O., Frick O., Herzberg C., Ehrenreich A., Heinzle E., Wittmann C., Stülke J. (2007). Transcriptional and metabolic responses of *Bacillus subtilis* to the availability of organic acids: Transcription regulation is important but not sufficient to account for metabolic adaptation. Appl. Environ. Microbiol..

[45] Çalík P., Çalík G., Özdamar T.H. (1998). Oxygen transfer effects in serine alkaline protease fermentation by *Bacillus licheniformis*: Use of citric acid as the carbon source. Enzym. Microb. Technol..

[46] Reeds P.J. (2000). Dispensable and indispensable amino acids for humans. J. Nutr..

[47] Tsuchiya K., Nishimura K., Iwahara M. (2003). Purification and characterization of glutamate decarboxylase from *Aspergillus oryzae*. Food Sci. Technol. Res..

[48] Margelis S., D’Souza C., Small A.J., Hynes M.J., Adams T.H., Davis M.A. (2001). Role of glutamine synthetase in nitrogen metabolite repression in *Aspergillus nidulans*. J. Bacteriol..

[49] Dhakal R., Bajpai V.K., Baek K.H. (2012). Production of GABA (γ-aminobutyric acid) by microorganisms: A review. Braz. J. Microbiol..

[50] Wernerman J. (2008). Clinical use of glutamine supplementation. J. Nutr..

[51] Murphy P.A., Barua K., Hauck C.C. (2002). Solvent extraction selection in the determination of isoflavones in soy foods. J. Chromatogr. B.

[52] Zhao D., Shah N.P. (2014). Changes in antioxidant capacity, isoflavone profile, phenolic and vitamin contents in soymilk during extended fermentation. LWT Food Sci. Technol..

[53] Day A.J., DuPont M.S., Ridley S., Rhodes M., Rhodes M.J., Morgan M.R., Williamson G. (1998). Deglycosylation of flavonoid and isoflavonoid glycosides by human small intestine and liver β-glucosidase activity. FEBS Lett..

[54] Izumi T., Piskula M.K., Osawa S., Obata A., Tobe K., Saito M., Kataoka S., Kubota Y., Kikuchi M. (2000). Soy isoflavone aglycones are absorbed faster and in higher amounts than their glucosides in humans. J. Nutr..

