# Supplementary material for: Metabolic Profiling-Based Evaluation of the Fermentative Behavior of Aspergillus oryzae and Bacillus subtilis for Soybean Residues Treated at Different Temperatures
Source: Foods. 2020 Jan 22;9(2):117. doi: 10.3390/foods9020117 (PMC7074079; doi:10.3390/foods9020117)
Supplement: Supplementary file 1 [file foods-09-00117-s001.pdf]

Supplementary Information

Article

Metabolic profiling–based evaluation of the fermentative behaviour of *Aspergillus oryzae* and *Bacillus subtilis* for soybean residues treated at different temperatures

Hyejin Hyeon [1,#], Cheol Woo Min [2,#], Keumok Moon [3], Jaeho Cha [3,4], Ravi Gupta [5], Sang Un Park [6], Sun Tae Kim [2,*], and Jae Kwang Kim [1,*]

[1] Division of Life Sciences, Incheon National University, Incheon, 22012, Republic of Korea
[2] Department of Plant Bioscience, Life and industry Convergence Research Institute, Pusan National University, Miryang, 50463, Republic of Korea
[3] Department of Microbiology, College of Natural Sciences, Pusan National University, Busan 46241, Republic of Korea
[4] Microbiological Resource Research Institute, Pusan National University, Busan 46241, Republic of Korea
[5] Department of Botany, School of Chemical and Life Science, Jamia Hamdard, New Delhi, 110062, India
[6] Department of Crop Science, Chungnam National University, 99 Daehak-ro, Yuseong-gu, Daejeon 34134, Republic of Korea
* Correspondence: kjkpj@inu.ac.kr (K.J.K.); stkim71@pusan.ac.kr (S.T.K.); Tel.: +82-32-835-8241 (K.J.K.); +82-55-350-5505 (S.T.K.); Fax: +82-32-835-0763 (K.J.K.); +82-55-350-5509 (S.T.K.)
[#] The two authors contributed equally to the article.

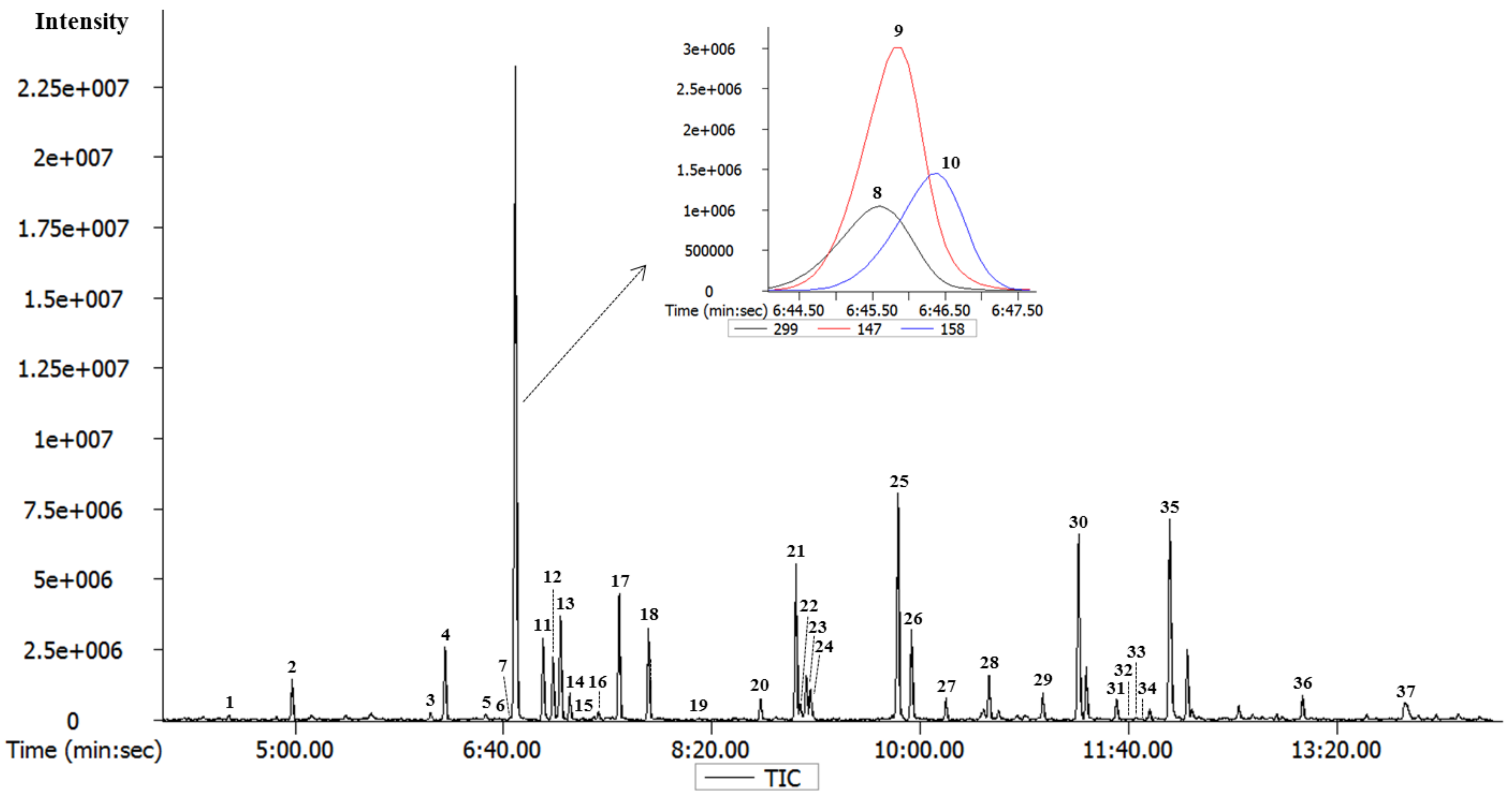

Figure S1. GC-TOF-MS chromatogram of *A.oryzae* (AO) fermented soybeans after extraction at 4 ºC. 1, Lactic acid; 2, Alanine; 3, Glycolic acid; 4, Valine; 5, Urea; 6. Serine-1; 7, Ethanolamine; 8, Phosphoric acid; 9, Glycerol; 10, Leucine; 11, Isoleucine; 12, Proline; 13, Glycine; 14, Succinic acid; 15, Glyceric acid; 16, Fumaric acid; 17, Serine-2; 18, Threonine; 19, β-Alanine; 20, Malic acid; 21, Aspartic acid; 22, Methionine; 23, Pyroglutamic acid; 24, γ-Aminobutyric acid; 25, Glutamic acid; 26, Phenylalanine; 27, Asparagine; 28, Ribitol (internal standard); 29, Glutamine; 30, Citric acid; 31, Fructose-1; 32, Fructose-2; 33, Galactose; 34, Glucose; 35, Mannitol; 36, Inositol; 37, Tryptophan.

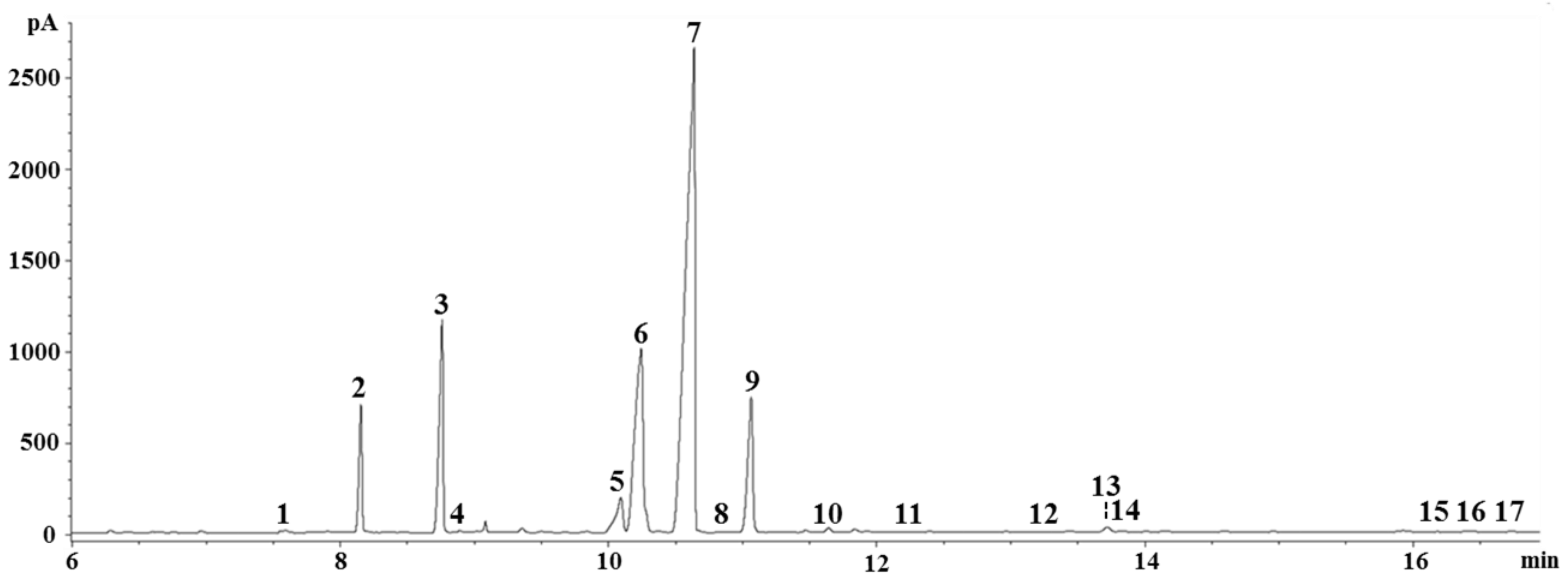

Figure S2. GC-FID chromatogram of *A.oryzae* (AO) fermented soybeans after extraction at 4 ºC. 1, C14:0; 2, Pentadecanoic acid (internal standard); 3, C16:0; 4, C16:1; 5, C18:0; 6, C18:1; 7, C18:2; 8, C18:3n6; 9, C18:3; 10, C20:0; 11, C20:3n6; 12, C20:5n3; 13, C22:0; 14, C22:1; 15, C22:5n3; 16, C22:6n3; 17, C24:0.

(A)

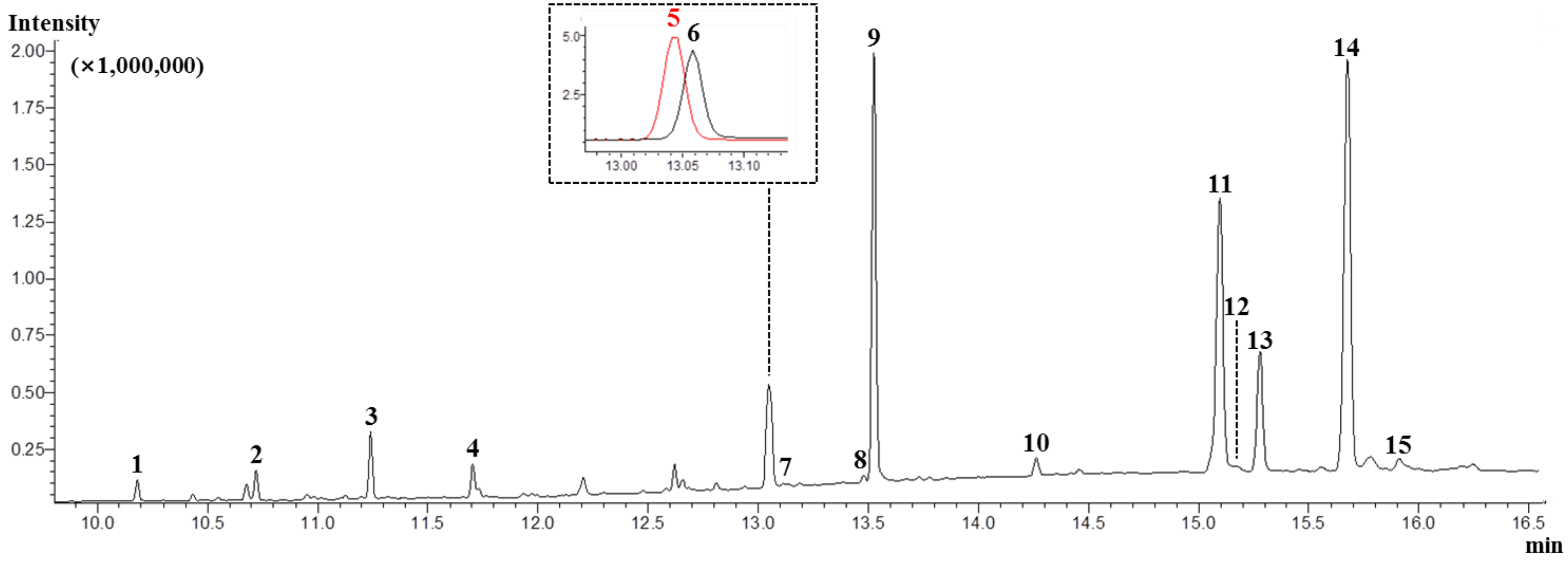

Figure S3. EIC (A) and selected ion monitoring (SIM) chromatogram (B) of secondary lipophilic compounds as TMS derivatives. GC-MS was used to analyze lipophilic metabolites of *A.oryzae* (AO) fermented soybeans after extraction at 4 ºC. The selected compounds are displayed in (B). Inverted triangles represents peak of target compound. 1, C20-ol; 2, C21-ol; 3, C22-ol; 4, C23-ol; 5, 5α-Cholestane (internal standard); 6, δ-Tocopherol; 7, C26-ol; 8, β-Tocopherol; 9, γ-Tocopherol; 10, α-Tocopherol; 11, Campesterol; 12, C30-ol; 13, Stigmasterol; 14, β-Sitosterol; 15, β-Amyrin.

(B)

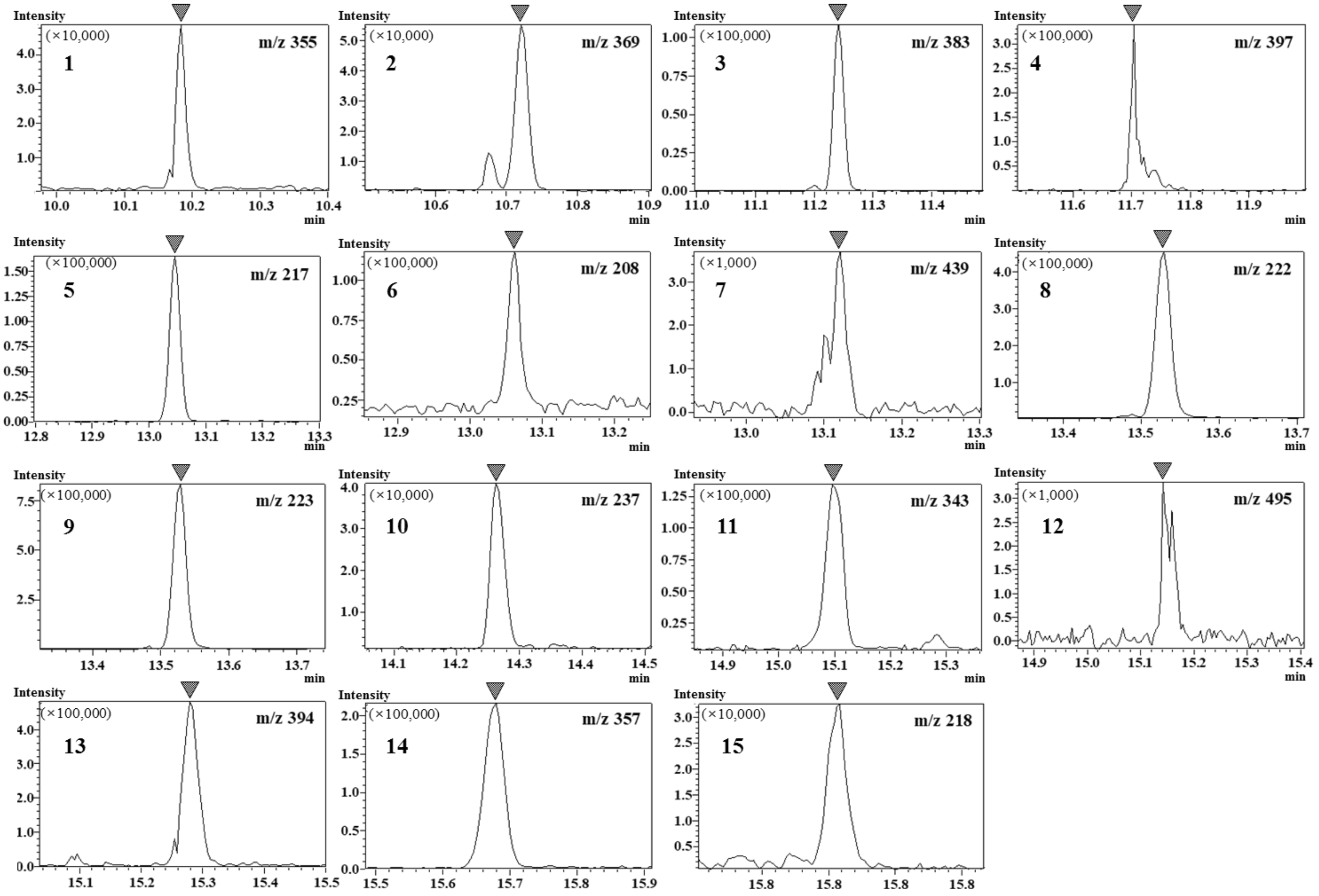

Figure S3. *(Continued).*

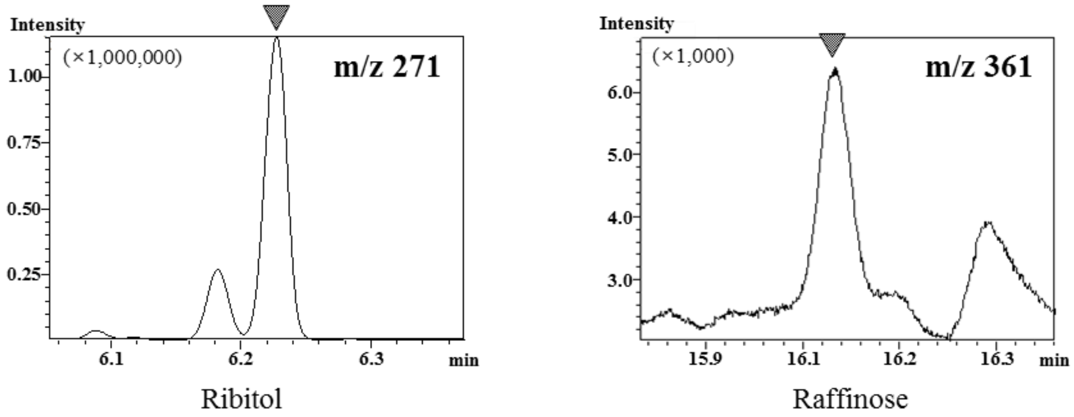

Figure S4. Representative selected ion monitoring (SIM) chromatogram of ribitol (internal standard) and raffinose in *A.oryzae* (AO) fermented soybeans after extraction at 4 ℃.

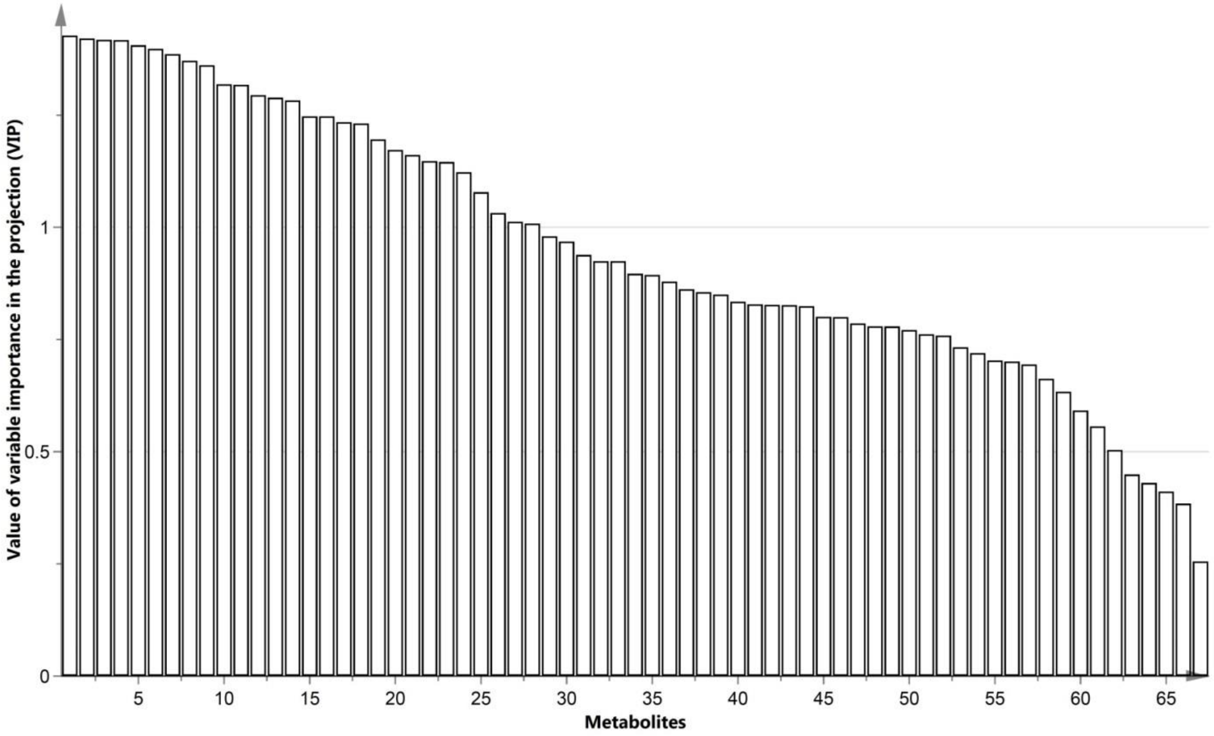

Figure S5. The influence of variables used to separate the extracted and then fermented soybeans by two types of microbes. Plot annotation 1, Ethanolamine; 2, γ-Aminobutyric acid; 3, Glycerol; 4, Fructose; 5, β-Alanine; 6, Malic acid; 7, C22:6n3; 8, Glutamine; 9, Inositol; 10, Citric acid; 11, Glyceric acid; 12, C18:3n6; 13, C22:0; 14, Campesterol; 15, Stigmasterol; 16, Asparagine; 17, β-Sitosterol; 18, Glucose; 19, Serine; 20, Pyroglutamic acid; 21, C14:0; 22, Mannitol; 23, Aspartic acid; 24, Phosphoric acid; 25, Glutamic acid; 26, α-Tocopherol; 27, Tryptophan; 28, Daidzein; 29, Phenylalanine; 30, C18:0; 31, Proline; 32, C16:1; 33, C22:1; 34, Methionine; 35, Lactic acid; 36, C22-ol; 37, C23-ol; 38, Urea; 39, Leucine; 40, γ-Tocopherol; 41, C20:5n3; 42, C16:0; 43, Genistein; 44, C18:1; 45, C18:3; 46, Valine; 47, Glycine; 48, C18:2; 49, Isoleucine; 50, Threonine; 51, Fumaric acid; 52, Succinic acid; 53, C30-ol; 54, Alanine; 55, β-Tocopherol; 56, β-Amyrin; 57, Glycolic acid; 58, C22:5n3; 59, Galactose; 60, C24:0; 61, Raffinose; 62, δ-Tocopherol; 63, C20-ol; 64, C20:3n6; 65, C21-ol; 66, C20:0; 67, C26-ol.

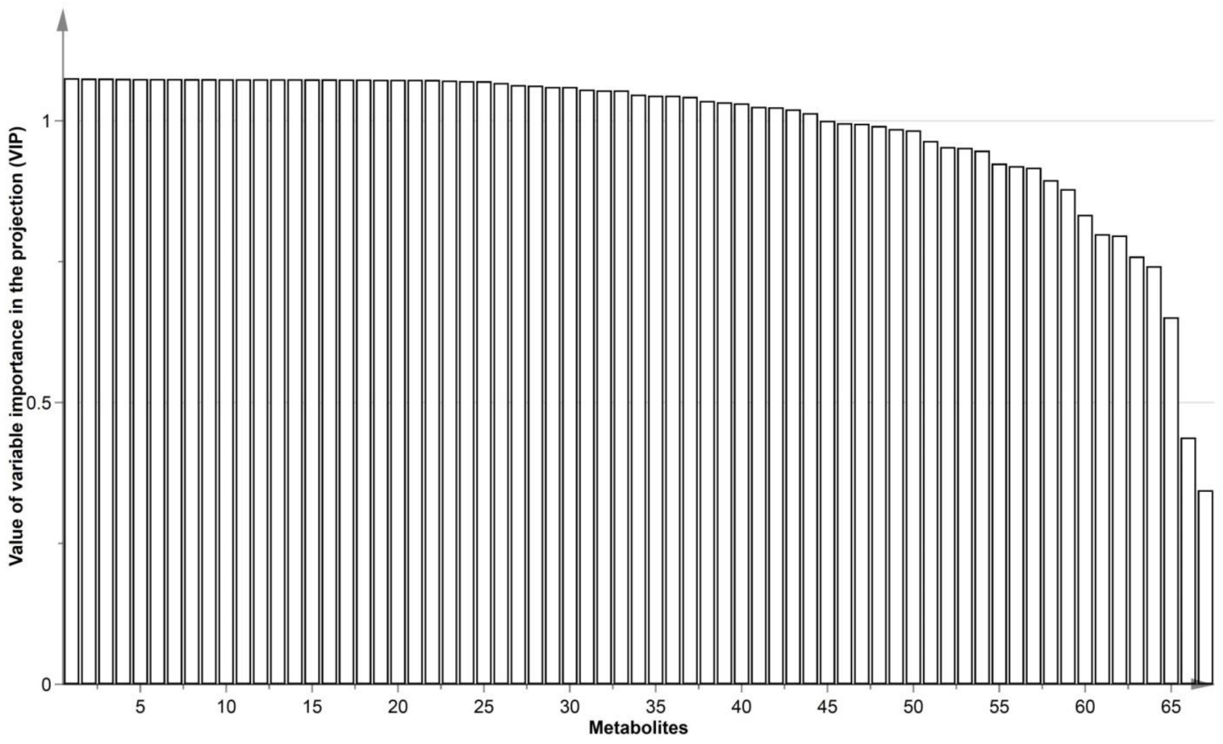

Figure S6. The influence of variables used to separate the soybeans extracted at 55 ºC by two types of microorganisms. Plot annotation 1, Genistein; 2, Mannitol; 3, Daidzein; 4, Citric acid; 5, Methionine; 6, Glutamine; 7, Valine; 8, Tryptophan; 9, Glycerol; 10, Leucine; 11, Phenylalanine; 12, Isoleucine; 13, Proline; 14, Threonine; 15, Glycine; 16, Lactic acid; 17, γ-Aminobutyric acid; 18, Urea; 19, Asparagine; 20, Ethanolamine; 21, Alanine; 22, Malic acid; 23, Aspartic acid; 24, Serine; 25, Glutamic acid; 26, Phosphoric acid; 27, Glycolic acid; 28, β-Alanine; 29, β-Sitosterol; 30, Campesterol; 31, C18:3; 32, C18:3n6; 33, Succinic acid; 34, Pyroglutamic acid; 35, Glucose; 36, γ-Tocopherol; 37, Inositol; 38, Galactose; 39, Fructose; 40, Fumaric acid; 41, Stigmasterol; 42, C22:0; 43, C22:6n3; 44, α-Tocopherol; 45, β-Tocopherol; 46, C18:2; 47, C18:1; 48, C20:5n3; 49, C24:0; 50, C14:0; 51, Raffinose; 52, C16:0; 53, β-Amyrin; 54, Glyceric acid; 55, C30-ol; 56, C22:1; 57, C20:0; 58, C22-ol; 59, C22:5n3; 60, C26-ol; 61, δ-Tocopherol; 62, C16:1; 63, C21-ol; 64, C20:3n6; 65, C20-ol; 66, C23-ol; 67, C18:0.

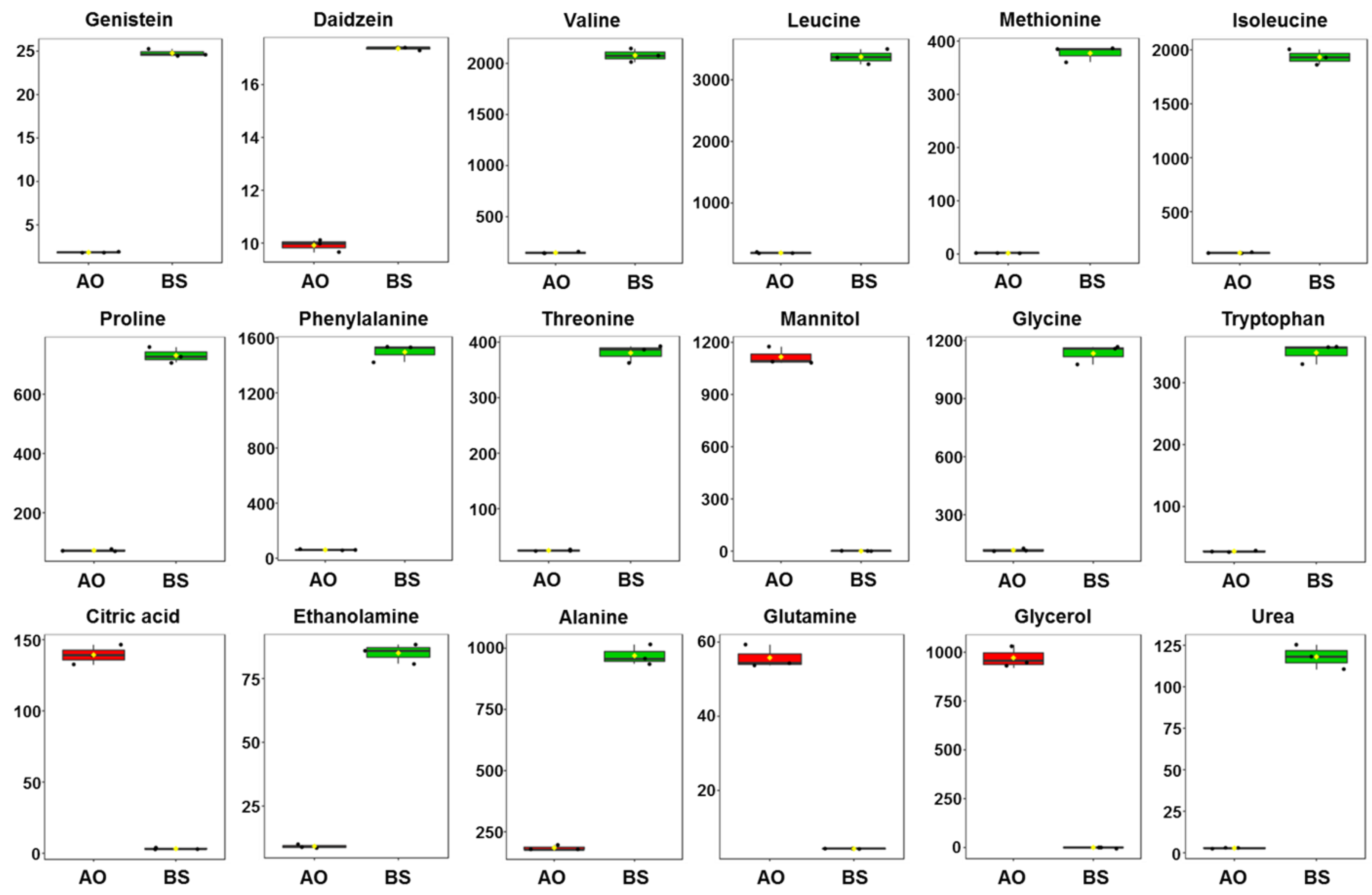

Figure S7. Box plots of significantly different metabolites between *A.oryzae* (AO) and *B.subtilis* (BS) fermented soybeans after extraction at 55 ºC. On the basis of variable importance in the projection (VIP) value of > 1.0 in the OPLS-DA model and the *P- value* ($P < 0.0001$) in the *t*-test for all metabolites, 26 metabolites were selected.

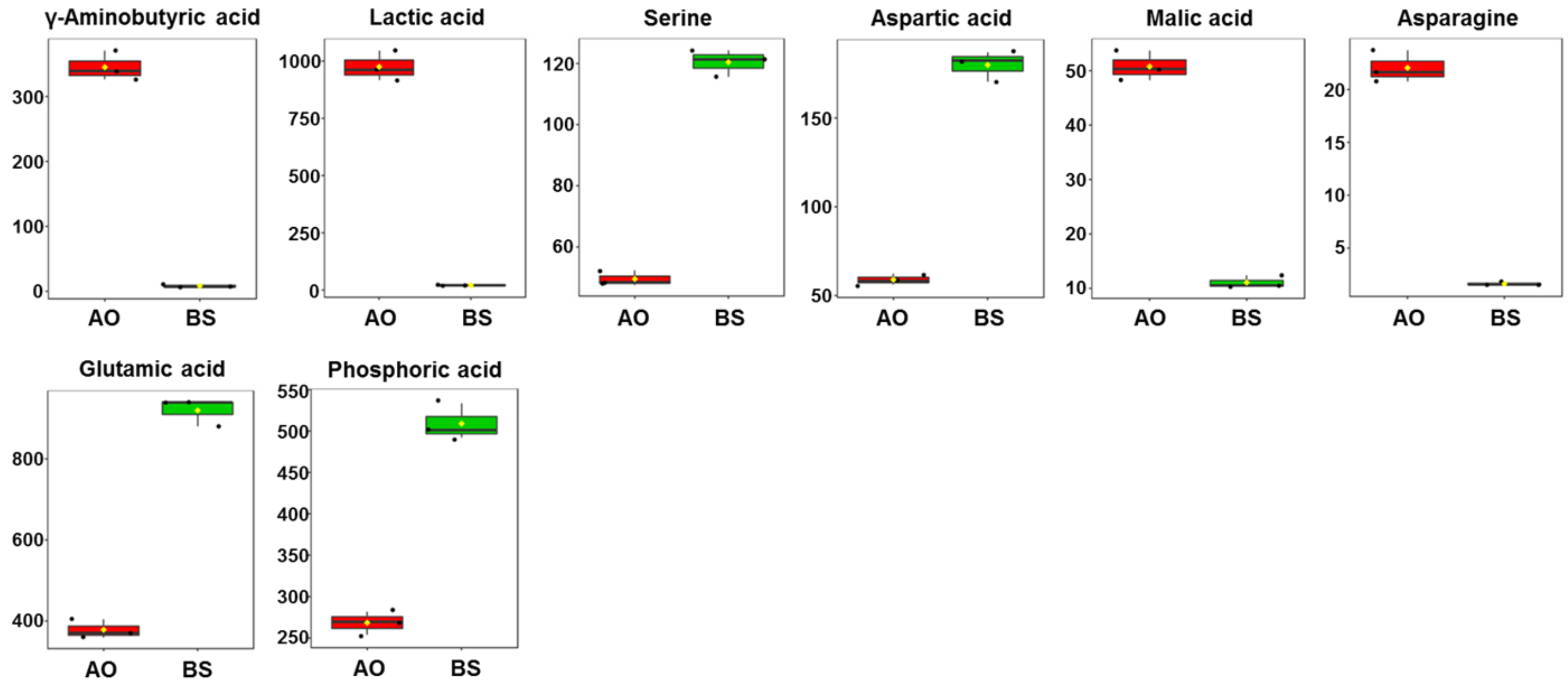

Figure S7. *(Continued)*.

Table S1. Relative retention times (RRT) and mass spectrometry data of identified hydrophilic compounds with GC-TOF-MS.

Compounds	[a] RT	[b] RRT	[c] Quantification ion	[d] Fragment ions
Lactic acid	04:28.1	0.424	147	117, 147, 191
Alanine	04:58.4	0.471	116	116, 147, 190
Glycolic acid	06:05.1	0.577	147	147, 177, 205
Valine	06:12.0	0.588	144	144, 156, 218
Urea	06:27.8	0.613	189	147, 171, 189
Serine-1	06:38.3	0.629	116	116, 132, 147
Ethanolamine	06:43.3	0.637	174	100, 147, 174
Phosphoric acid	06:45.2	0.640	299	299
Glycerol	06:45.7	0.641	147	147, 177, 205
Leucine	06:46.2	0.642	158	102, 147, 158
Isoleucine	06:59.0	0.662	158	147, 158, 218
Proline	07:03.5	0.669	142	142, 158, 216
Glycine	07:07.2	0.675	174	147, 174, 248
Succinic acid	07:11.7	0.682	147	129, 147, 247
Glyceric acid	07:18.0	0.692	147	133, 147, 189
Fumaric acid	07:32.1	0.714	245	143, 147, 245
Serine-2	07:35.5	0.720	204	204, 278, 306
Threonine	07:49.4	0.742	219	117, 218, 291
β-Alanine	08:14.4	0.781	174	147, 174, 248
Malic acid	08:43.3	0.827	147	147, 233, 245
Aspartic acid	09:00.3	0.854	100	100, 147, 232
Methionine	09:02.4	0.857	176	128, 147, 176
Pyroglutamic acid	09:05.1	0.861	156	147, 156, 230
γ-Aminobutyric acid	09:07.1	0.864	174	147, 174, 304
Glutamic acid	09:49.1	0.931	246	128, 156, 246
Phenylalanine	09:55.6	0.941	218	100, 192, 218
Asparagine	10:12.5	0.968	116	116, 132, 231
Ribitol (Internal standard)	10:32.9	1.000	217	103, 147, 217
Glutamine	10:58.9	1.041	156	147, 156, 245
Citric acid	11:15.8	1.068	273	147, 273, 347
Fructose-1	11:35.2	1.098	103	103, 147, 217
Fructose-2	11:39.3	1.105	103	103, 147, 217
Galactose	11:43.5	1.112	147	147, 205, 319
Glucose	11:46.1	1.116	160	147, 160, 205
Mannitol	11:59.4	1.137	319	147, 205, 319
Inositol	13:03.4	1.238	305	147, 217, 305
Tryptophan	13:53.3	1.317	202	202, 219, 348

[a] Retention time (min:sec)

[b] Relative retention time (retention time of the analyte/retention time of the IS)

[c] Specific mass ion used for quantification

[d] MS fragment ions with large characteristic intensities.

Table S2. Relative retention times (RRT) of fatty acid methyl esters (FAME) mixture and other fatty acids for quantification with GC-FID.

Compounds	C	[a] RT	[b] RRT	Area	Weight %	ppm	μg	Ratio
Myristic acid	C14:0	7.559	0.926	126.2	8	80	8	0.071
Pentadecanoic acid (Internal standard)	C15:0	8.166	1.000	1789.7		1000	100	1.000
Palmitic acid	C16:0	8.734	1.070	178.8	11	110	11	0.100
Palmitoleic acid	C16:1	8.890	1.089	76.3	5	50	5	0.043
Stearic acid	C18:0	10.034	1.229	127.5	8	80	8	0.071
Oleic acid	C18:1	10.185	1.247	78.4	5	50	5	0.044
Linoleic acid	C18:2	10.523	1.289	59.7	5	50	5	0.033
γ-Linolenic acid	C18:3n6	10.760	1.318	204.1		100	10	0.114
α-Linolenic acid	C18:3	11.029	1.351	42.4	5	50	5	0.024
Arachidic acid	C20:0	11.663	1.428	136.3	8	80	8	0.076
dihomo-γ-Linolenic acid	C20:3n6	12.585	1.541	158.3		100	10	0.088
Eicosapentaenoic acid	C20:5n3	13.529	1.657	128.4		100	10	0.072
Behenic acid	C22:0	13.756	1.685	132.1	8	80	8	0.074
Erucic acid	C22:1	14.016	1.716	79.4	5	50	5	0.044
Eicosapentaenoic acid	C22:5n3	16.224	1.987	139.7		100	10	0.078
Docosahexaenoic acid	C22:6n3	16.469	2.017	134.7		100	10	0.075
Lignoceric acid	C24:0	16.667	2.041	130.9	8	80	8	0.073

[a] Retention time (min)

[b] Relative retention times (retention time of analyte/retention time of C15:0)

Table S3. Relative retention times (RRT) and mass spectrometry data of secondary lipophilic compounds with GC-MS.

Compounds	[a] RT	[b] RRT	$[M]^+$	[c] Quantification ions	[d] Fragment ions [m/z (%)]
C20-ol (Eicosanol)	10.302	0.781	370 (0)	355	355 [100]
C21-ol (Heneicosanol)	10.843	0.822	384 (0)	369	369 [100]
C22-ol (Docosanol)	11.364	0.861	398 (0)	383	383 [100]
C23-ol (Tricosanol)	11.830	0.897	411 (0)	397	397 [100]
δ-Tocopherol	13.195	1.000	474 (100)	208	208 [90], 249 [25]
β-Tocopherol	13.625	1.033	489 (100)	222	263 [13], 222 [87]
γ-Tocopherol	13.673	1.036	488 (60)	223	263 [10], 223 [100]
α-Tocopherol	14.442	1.095	503 (90)	237	277 [8], 237 [100]
Campesterol	15.318	1.161	473 (0)	343	382 [38], 367 [23], 343 [50], 255 [18], 129 [100]
C30-ol (Triacontanol)	15.328	1.162	510 (0)	495	496 [100] ,495 [60]
Stigmasterol	15.508	1.175	484 (23)	394	394 [38], 355 [15], 255 [52], 129 [100]
β-Sitosterol	15.925	1.207	487 (17)	357	396 [42], 357 [44], 255 [17], 129 [100]
β-Amyrin	16.175	1.226	499 (0)	218	218 [100], 203 [42], 189 [26]

[a] Retention time (min)

[b] Relative retention time (retention time of analyte/retention time of 5α-cholestane)

[c] Specific mass ion used for quantification

[d] MS fragment ions with large characteristic intensities

Table S4. Composition and content (ratio/g) of hydrophilic compounds in soybeans with GC-TOF-MS analysis

	[a]AO			BS		
Compounds	[b]4 ℃	25 ℃	55 ℃	4 ℃	25 ℃	55 ℃
				Amino acids		
Alanine	510.59 ± 53.79	457.02 ± 52.42	184.26 ± 10.38	10.70 ± 2.93	11.68 ± 0.66	969.32 ± 41.59
Asparagine	92.00 ± 5.05	112.71 ± 2.81	22.05 ± 1.52	[c]ND	ND	1.62 ± 0.18
Aspartic acid	423.89 ± 31.84	248.17 ± 2.17	58.93 ± 3.17	2.18 ± 0.21	ND	179.89 ± 8.40
Glutamic acid	1187.98 ± 97.55	908.72 ± 1.10	378.45 ± 23.66	41.48 ± 1.51	27.60 ± 0.36	919.50 ± 34.03
Glutamine	134.26 ± 5.61	140.19 ± 5.84	55.79 ± 3.09	ND	ND	4.28 ± 0.04
Glycine	719.68 ± 62.02	531.61 ± 3.31	117.16 ± 7.08	7.67 ± 0.91	2.91 ± 0.13	1133.19 ± 49.88
Isoleucine	698.64 ± 64.62	547.14 ± 5.88	117.52 ± 5.29	136.48 ± 32.64	89.27 ± 1.36	1932.07 ± 71.79
Leucine	1115.73 ± 90.01	869.30 ± 10.17	191.58 ± 10.20	471.28 ± 133.36	382.13 ± 3.84	3371.67 ± 122.62
Methionine	89.12 ± 7.72	61.13 ± 0.32	1.69 ± 0.20	58.02 ± 3.19	21.39 ± 0.28	377.12 ± 14.76
Phenylalanine	455.15 ± 39.26	331.76 ± 3.32	60.81 ± 3.95	404.40 ± 27.06	261.36 ± 3.04	1495.74 ± 63.52
Proline	893.97 ± 73.96	534.87 ± 3.36	72.59 ± 3.35	139.81 ± 43.13	54.22 ± 0.91	730.64 ± 26.36
Pyroglutamic acid	597.82 ± 67.72	461.26 ± 11.24	131.30 ± 10.53	82.93 ± 14.64	79.56 ± 2.53	252.89 ± 24.45
Serine	613.55 ± 53.33	447.24 ± 1.94	49.53 ± 2.52	0.93 ± 0.29	ND	120.42 ± 4.45
Threonine	211.77 ± 17.54	155.05 ± 0.25	24.77 ± 1.48	4.63 ± 0.37	1.08 ± 0.15	380.62 ± 15.94
Tryptophan	187.86 ± 11.09	132.33 ± 1.57	27.73 ± 1.27	169.84 ± 8.92	101.92 ± 1.34	348.45 ± 15.92
Valine	729.84 ± 59.56	576.69 ± 9.58	148.13 ± 8.53	180.99 ± 57.41	188.43 ± 5.06	2078.26 ± 65.36
β-Alanine	4.60 ± 0.36	4.59 ± 0.06	4.61 ± 0.24	1.07 ± 0.15	ND	2.44 ± 0.24
γ-Aminobutyric acid	212.47 ± 18.12	170.46 ± 0.63	345.07 ± 22.89	9.59 ± 0.04	ND	8.30 ± 2.49

Table S4. *(Continued).*

			Organic acids			
Citric acid	540.56 ± 33.29	453.09 ± 4.13	139.28 ± 6.94	23.07 ± 0.99	6.30 ± 1.56	3.20 ± 0.64
Fumaric acid	10.37 ± 0.48	8.17 ± 0.36	4.99 ± 0.35	5.85 ± 0.74	7.76 ± 0.24	3.68 ± 0.08
Glyceric acid	7.30 ± 0.43	4.34 ± 0.25	4.39 ± 0.24	ND	ND	3.34 ± 0.49
Glycolic acid	95.84 ± 6.55	76.64 ± 1.99	35.03 ± 2.01	96.51 ± 7.90	102.80 ± 1.71	68.95 ± 3.96
Lactic acid	33.68 ± 3.55	33.30 ± 2.74	974.69 ± 66.22	105.24 ± 8.63	26.60 ± 4.04	20.72 ± 2.10
Malic acid	122.92 ± 8.80	98.34 ± 0.73	50.76 ± 2.71	5.45 ± 0.11	5.48 ± 0.45	11.07 ± 1.16
Succinic acid	299.65 ± 23.72	317.02 ± 5.47	459.38 ± 25.51	536.39 ± 27.86	287.63 ± 2.82	695.66 ± 30.64
Urea	20.66 ± 1.60	12.29 ± 0.44	2.79 ± 0.28	3.78 ± 0.31	8.16 ± 0.42	118.05 ± 7.37
			Sugars and sugar alcohols			
Fructose	6.55 ± 0.37	4.56 ± 0.37	2.93 ± 0.81	ND	ND	ND
Galactose	6.49 ± 0.28	6.93 ± 0.22	2.52 ± 0.68	17.6 ± 0.78	12.47 ± 0.72	ND
Glucose	0.96 ± 0.31	1.21 ± 0.93	0.84 ± 0.19	ND	ND	ND
Glycerol	2144.64 ± 143.41	1930.36 ± 83.72	971.17 ± 60.96	ND	ND	ND
Inositol	44.57 ± 3.44	41.28 ± 0.57	42.01 ± 2.34	11.78 ± 0.87	2.80 ± 0.15	27.95 ± 2.33
Mannitol	236.88 ± 19.10	264.85 ± 5.24	1115.78 ± 51.23	ND	ND	ND
			Others			
Ethanolamine	17.87 ± 1.71	12.19 ± 0.33	9.21 ± 0.76	52.86 ± 4.90	52.88 ± 1.05	85.03 ± 3.88
Phosphoric acid	865.90 ± 53.20	527.13 ± 21.53	268.47 ± 14.13	141.76 ± 6.46	96.55 ± 0.97	509.00 ± 21.96

Each value is the mean of three replications ± standard deviation.

[a] AO, *Aspergillus oryzae* fermented soybeans after water extraction; BS, *Bacillus subtilis* fermented soybeans after water extraction

[b] Water extraction temperatures of soybean substrates

[c] ND = not detected.

Table S5. Composition and content (μg/mg) of fatty acids in soybeans with GC-FID analysis

Compounds	[a] AO fermentation			BS fermentation		
	[b] 4 ℃	25 ℃	55 ℃	4 ℃	25 ℃	55 ℃
			Fatty acids			
C14:0	0.55 ± 0.06	0.56 ± 0.03	0.49 ± 0.03	0.64 ± 0.02	0.69 ± 0.06	0.61 ± 0.05
C16:0	30.14 ± 1.98	28.18 ± 1.71	22.21 ± 0.51	25.13 ± 1.01	25.03 ± 1.21	26.98 ± 2.20
C16:1	0.25 ± 0.06	0.24 ± 0.05	0.22 ± 0.04	0.18 ± 0.03	0.22 ± 0.04	0.16 ± 0.03
C18:0	9.65 ± 0.66	9.26 ± 0.50	9.67 ± 0.23	8.06 ± 0.38	8.02 ± 0.43	9.47 ± 1.17
C18:1	55.18 ± 4.13	52.56 ± 3.73	44.42 ± 1.36	49.23 ± 1.63	49.13 ± 3.02	57.36 ± 4.64
C18:2	216.01 ± 15.77	200.13 ± 14.60	150.70 ± 4.26	183.18 ± 6.46	183.75 ± 10.68	199.86 ± 17.75
C18:3n6	0.12 ± 0.03	0.13 ± 0.03	0.13 ± 0.01	0.08 ± 0.01	0.08 ± 0.01	0.07 ± 0.01
C18:3	45.28 ± 3.19	41.67 ± 3.42	22.11 ± 0.63	38.83 ± 1.40	38.60 ± 2.19	41.88 ± 3.60
C20:0	0.78 ± 0.07	0.77 ± 0.02	0.78 ± 0.01	0.73 ± 0.09	0.77 ± 0.07	0.84 ± 0.03
C20:3n6	0.17 ± 0.02	0.16 ± 0.01	0.16 ± 0.01	0.16 ± 0.01	0.17 ± 0.02	0.17 ± 0.02
C20:5n3	0.49 ± 0.16	0.48 ± 0.04	0.45 ± 0.03	0.42 ± 0.01	0.44 ± 0.04	0.33 ± 0.04
C22:0	1.37 ± 0.28	1.32 ± 0.10	1.33 ± 0.01	1.09 ± 0.04	1.06 ± 0.06	1.03 ± 0.08
C22:1	0.23 ± 0.04	0.21 ± 0.02	0.23 ± 0.02	0.17 ± 0.01	0.21 ± 0.04	0.19 ± 0.02
C22:5n3	0.08 ± 0.01	0.12 ± 0.03	0.21 ± 0.07	0.14 ± 0.03	0.12 ± 0.02	0.11 ± 0.01
C22:6n3	0.59 ± 0.07	0.56 ± 0.06	0.66 ± 0.04	0.38 ± 0.03	0.37 ± 0.03	0.44 ± 0.05
C24:0	0.14 ± 0.02	0.10 ± 0.03	0.10 ± 0.01	0.13 ± 0.01	0.14 ± 0.02	0.13 ± 0.01

Each value is the mean of three replications ± standard deviation.

[a] AO, *Aspergillus oryzae* fermented soybeans after water extraction; BS, *Bacillus subtilis* fermented soybeans after water extraction

[b] Water extraction temperatures of soybean substrates

Table S6. Composition and content (μg/g) of secondary lipophilic compounds in soybeans with GC-MS analysis

Compounds	[a] AO fermentation			BS fermentation		
	[b] 4 ℃	25 ℃	55 ℃	4 ℃	25 ℃	55 ℃
Policosanols						
C20-ol	10.07 ± 0.19	9.69 ± 0.33	9.41 ± 0.73	9.49 ± 0.51	9.66 ± 0.26	9.73 ± 0.32
C21-ol	7.27 ± 0.08	7.34 ± 0.46	7.02 ± 0.22	6.69 ± 0.32	7.11 ± 0.29	7.57 ± 0.82
C22-ol	26.21 ± 2.13	26.81 ± 1.88	25.36 ± 1.06	26.84 ± 1.09	29.39 ± 0.85	28.58 ± 2.66
C23-ol	3.39 ± 0.62	2.78 ± 0.20	3.23 ± 0.18	1.75 ± 0.22	1.81 ± 0.35	3.47 ± 0.56
C26-ol	7.11 ± 0.08	7.31 ± 0.26	7.42 ± 0.02	7.18 ± 0.19	6.89 ± 0.37	7.49 ± 0.05
C30-ol	18.02 ± 3.25	10.37 ± 1.37	9.28 ± 1.07	13.24 ± 1.85	7.12 ± 0.78	12.76 ± 1.79
Tocopherols						
α-Tocopherol	14.09 ± 0.30	13.57 ± 1.06	12.08 ± 1.35	13.77 ± 0.78	14.52 ± 0.50	16.76 ± 1.56
β-Tocopherol	6.85 ± 0.11	6.76 ± 0.08	6.66 ± 0.17	6.66 ± 0.34	6.80 ± 0.21	7.22 ± 0.10
γ-Tocopherol	73.27 ± 3.20	72.72 ± 6.00	65.59 ± 7.05	65.82 ± 12.60	75.99 ± 1.40	96.87 ± 1.88
δ-Tocopherol	12.29 ± 0.29	12.09 ± 0.36	11.94 ± 0.41	12.20 ± 0.44	12.25 ± 0.41	12.34 ± 0.12
Sterols						
Campesterol	491.00 ± 13.87	408.12 ± 25.96	540.00 ± 25.19	373.48 ± 4.88	260.94 ± 20.15	349.24 ± 16.35
Stigmasterol	239.31 ± 8.25	189.11 ± 12.73	258.98 ± 26.77	174.06 ± 7.41	127.82 ± 5.53	167.10 ± 10.70
β-Sitosterol	355.83 ± 7.61	300.59 ± 25.49	381.07 ± 13.89	266.35 ± 18.24	207.03 ± 10.31	286.82 ± 4.03
Others						
β-Amyrin	11.81 ± 0.44	11.28 ± 0.48	11.51 ± 0.30	11.68 ± 0.66	11.68 ± 0.60	12.53 ± 0.52

Each value is the mean of three replications ± standard deviation.

[a] AO, *Aspergillus oryzae* fermented soybeans after water extraction; BS, *Bacillus subtilis* fermented soybeans after water extraction

[b] Water extraction temperatures of soybean substrates

Table S7. Composition and content of raffinose (μg/mg) and isoflavonse (Area %) with GC-MS and UPLC-QTOF-MS analysis

	[a] AO fermentation			BS fermentation		
Compounds	[b] 4 ℃	25 ℃	55 ℃	4 ℃	25 ℃	55 ℃
			Raffinose			
Raffinose	0.10 ± 0.01	0.09 ± 0.01	0.10 ± 0.01	0.13 ± 0.01	0.10 ± 0.01	0.09 ± 0.01
			Isoflavones			
Daidzein	19.09 ± 3.92	20.73 ± 2.00	9.91 ± 0.23	7.57 ± 0.20	7.82 ± 0.43	17.36 ± 0.06
Genistein	22.52 ± 4.29	23.26 ± 5.24	1.84 ± 0.06	6.57 ± 0.64	8.43 ± 0.42	24.77 ± 0.43

Each value is the mean of three replications ± standard deviation.

[a] AO, *Aspergillus oryzae* fermented soybeans after water extraction; BS, *Bacillus subtilis* fermented soybeans after water extraction

[b] Water extraction temperatures of soybean substrates
